# The utility of Next Generation Sequencing for molecular diagnostics in Rett syndrome

**DOI:** 10.1038/s41598-017-11620-3

**Published:** 2017-09-25

**Authors:** Silvia Vidal, Núria Brandi, Paola Pacheco, Edgar Gerotina, Laura Blasco, Jean-Rémi Trotta, Sophia Derdak, Maria del Mar O’Callaghan, Àngels Garcia-Cazorla, Mercè Pineda, Judith Armstrong, Rett Working Group, Francisco Javier Aguirre, Montserrat Aleu, Xènia Alonso, Mercè Alsius, Maria Inmaculada Amorós, Guillermo Antiñolo, Lourdes Aquino, Carmen Arellano, Gema Arriola, Rosa Arteaga, Neus Baena, Montserrat Barcos, Nuria Belzunces, Susana Boronat, Tomás Camacho, Jaume Campistol, Miguel del Campo, Andrea Campo, Ramon Cancho, Ramon Candau, Ignacio Canós, María del Carmen Carrascosa, Francisco Carratalá-Marco, Jovaní Casano, Pedro Castro, Ana Cobo, Jaime Colomer, David Conejo, Maria José Corrales, Rocío Cortés, Gabriel Cruz, Gábor Csányi, María Teresa de Santos, María de Toledo, Mireia Del Toro, Rosario Domingo, Anna Duat, Rosario Duque, Ana María Esparza, Rosa Fernández, Maria Carme Fons, Ana Fontalba, Enrique Galán, Pia Gallano, María José Gamundi, Pedro Luis García, María del Mar García, María García-Barcina, María Jesús Garcia-Catalan, Sixto García-Miñaur, Juan Jose Garcia-Peñas, María Teresa García-Silva, Rosa Gassio, Esther Geán, Belén Gil, Sarenur Gökben, Luis Gonzalez, Veronica Gonzalez, Julieta Gonzalez, Gloria González, Encarna Guillén, Miriam Guitart, Montserrat Guitet, Juan Manuel Gutierrez, Eva Gutiérrez, Jose Luís Herranz, Gemma Iglesias, Iva Karacic, Carlos H. Lahoz, José Ignacio Lao, Pablo Lapunzina, María Jesús Lautre-Ecenarro, María Dolores Lluch, Laura López, Asunción López-Ariztegui, Alfons Macaya, Rosario Marín, Charles M. Lourenço Marquez, Elena Martín, Beatriz Martínez, Eduardo Martínez-Salcedo, María José Mas, Gonzalo Mateo, Pilar Mendez, Amparo Morant Jimenez, Sira Moreno, Fernando Mulas, Juan Narbona, Andrés Nascimento, Manuel Nieto, Tania Fabiola Nunes, Núria Núñez, María Obón, Ignacio Onsurbe, Carlos Ignacio Ortez, Emilio Orts, Francisco Martinez, Rafael Parrilla, Samuel Ignacio Pascual, Ana Patiño, Maria Pérez-Poyato, Belén Pérez-Dueñas, Pilar Póo, Eliodoro Puche, Feliciano Ramos, Miquel Raspall, Ana Roche, Susana Roldan, Jordi Rosell, Cesar Ruiz, María Luz Ruiz-Falcó, Maria Eugenia Russi, Jordi Samarra, Victoria San Antonio, Ivan Sanchez, Xavier Sanmartin, Ana Sans, Alfredo Santacana, Sabine Scholl-Bürgi, Nuria Serrano, Mercedes Serrano, Pilar Martin-Tamayo, Adrián Tendero, Jaime Torrents, Diego Tortosa, Emma Triviño, Ledia Troncoso, Eulalia Turrón, Pilar Vázquez, Carlos Vázquez, Ramón Velázquez, Clara Ventura, Alfonso Verdú, Anna Vernet, M. Tomás Vila, Cristina Villar

**Affiliations:** 1 Molecular and Genetics Medicine Section, Hospital Sant Joan de Déu, Barcelona, Spain; 2 Facultat de Medicina, Universitat de Barcelona, Barcelona, Spain; 3 Centro Nacional de Análisis Genómica (CNAG-CRG), Center for Genomic Regulation, Barcelona Institute of Science and Technology (BIST), Barcelona, Spain; 4 Neurology Service, Hospital Sant Joan de Déu, Barcelona, Spain; 5 Institut de Recerca Pediàtrica Hospital Sant Joan de Déu, Barcelona, Spain; 6 CIBER-ER (Biomedical Network Research Center for Rare Diseases), Instituto de Salud Carlos III, Madrid, Spain; 7 Hospital Torrecardenas, Almeria, Spain; 8 Consorcio Hospital General Universitario de Valencia, Valencia, Spain; 9 Hospital Universitari Dr. Josep Trueta, Girona, Spain; 10 Hospital Punta Europa, Cadiz, Spain; 11 Hospital Universitario Virgen del Rocio, Sevilla, Spain; 12 Hospital de Mataró, Mataro, Spain; 13 Consorci Sanitari, Terrassa, Spain; 14 Hospital universitario de Guadalajara, Guadalajara, Spain; 15 Hospital Universitario Marqués de Valdecilla, Santander, Spain; 16 Hospital de Sabadell, Barcelona, Spain; 17 Hospital Universitario Reina Sofía, Cordoba, Spain; 18 Balagué Center, Barcelona, Spain; 19 Hospital de la Vall d’Hebrón, Barcelona, Spain; 20 Lema & Bandin Laboratorios, Vigo, Spain; 21 Hospital Universitario Río Hortega, Valladolid, Spain; 22 Hospital Universitario Doctor Peset, Valencia, Spain; 23 Hospital General Universitario de Albacete, Albacete, Spain; 24 Hospital Universitari San Juan, Alicante, Spain; 25 Hospital Universitario General de Castellón, Castellon de la Plana, Spain; 26 Hospital Gregorio Marañón, Madrid, Spain; 27 Hospital de Donostia, San Sebastian, Spain; 28 Complejo asistencial, Burgos, Spain; 29 Hospital General Mancha Centro, Ciudad Real, Spain; 30 Hospital San Borja Arriaran, Santiago, Chile; 31 Hospital Universitario de Valme, Sevilla, Spain; 32 Hospital de la Santa Creu i Sant Pau, Barcelona, Spain; 33 Hospital de Fuenlabrada, Madrid, Spain; 34 Hospital Universitario Severo Ochoa, Madrid, Spain; 35 Hospital Infantil de La Arrixaca, Murcia, Spain; 36 Hospital Infantil Universitario Niño Jesús, Madrid, Spain; 37 Hospital Universitario Nuestra Señora de la Candelaria, Santa Cruz de Tenerife, Spain; 38 Hospital Universitario de Getafe, Madrid, Spain; 39 Hospital Materno-Infantil de Badajoz, Badajoz, Spain; 40 Hospital Virgen de la Salud, Toledo, Spain; 41 Hospital Cormarcal de Figueres, Girona, Spain; 42 Hospital de Basurto, Bilbao, Spain; 43 Hospital La Paz, Madrid, Spain; 44 Hospital Universitario 12 de Octubre, Madrid, Spain; 45 Ege Ünŭversŭtesŭ Tip Fakültesŭ Pedŭatrŭ AD, Uzmur, Turkey; 46 Hospital General de Granollers, Barcelona, Spain; 47 Hospital Clínico Universitario de Valladolid, Valladolid, Spain; 48 Hospital Virgen de la Luz, Cuenca, Spain; 49 Clinical Hospital Center Zagreb, Zagreb, Croatia; 50 Hospital Central Asturias, Asturias, Spain; 51 Laboratorio Echevarne, Barcelona, Spain; 52 Hospital Clínico San Carlos, Madrid, Spain; 53 Hospital Universitario Virgen Macarena, Sevilla, Spain; 54 Hospital de Cruces, Bilbao, Spain; 55 Hospital Universitario Puerta del Mar, Cadiz, Spain; 56 Medical Genetics Service, Clinics Hospital of Ribeirão Preto, University of São Paulo, Sao Paulo, Brazil; 57 Hospital General Universitario de Alicante, Alicante, Spain; 58 Hospital Joan XXIII, Tarragona, Spain; 59 Centro privado, Valencia, Spain; 60 Hospital Virgen del Camino, Pamplona, Spain; 61 Instituto Valenciano de Neurociencias, Valencia, Spain; 62 Clínica Universitaria de Pamplona, Pamplona, Spain; 63 Complejo Hospitalario de Jaén, Jaen, Spain; 64 Hospital de Navarra, Navarra, Spain; 65 Hospital Clínico Universitario Lozano Blesa, Zaragoza, Spain; 66 Hospital Universitario Virgen de las Nieves, Granada, Spain; 67 Hospital Son Dureta, Palma de Mallorca, Spain; 68 Hospital Costa del Sol, Malaga, Spain; 69 Hospital General de Vic, Barcelona, Spain; 70 Complejo Hospitalario Universitario Insular, Las Palmas de Gran Canaria, Spain; 71 Medizinische Universität Innsbruck, Innsbruck, Austria; 72 Althaia, Manresa, Spain; 73 Reference Laboratory, Barcelona, Spain; 74 Catlab, Barcelona, Spain; 75 Hospital Francesc De Borja, Valencia, Spain

**Keywords:** Genetics, Neuroscience

## Abstract

Rett syndrome (RTT) is an early-onset neurodevelopmental disorder that almost exclusively affects girls and is totally disabling. Three genes have been identified that cause RTT: *MECP2*, *CDKL5* and *FOXG1*. However, the etiology of some of RTT patients still remains unknown. Recently, next generation sequencing (NGS) has promoted genetic diagnoses because of the quickness and affordability of the method. To evaluate the usefulness of NGS in genetic diagnosis, we present the genetic study of RTT-like patients using different techniques based on this technology. We studied 1577 patients with RTT-like clinical diagnoses and reviewed patients who were previously studied and thought to have RTT genes by Sanger sequencing. Genetically, 477 of 1577 patients with a RTT-like suspicion have been diagnosed. Positive results were found in 30% by Sanger sequencing, 23% with a custom panel, 24% with a commercial panel and 32% with whole exome sequencing. A genetic study using NGS allows the study of a larger number of genes associated with RTT-like symptoms simultaneously, providing genetic study of a wider group of patients as well as significantly reducing the response time and cost of the study.

## Introduction

Rett syndrome (RTT; MIM# 312750) is a neurodevelopmental disorder of early onset that affects girls almost exclusively. RTT was originally described in the 1960s by Andreas Rett^1^. This syndrome is first recognized in infancy with a period of apparently normal development (up to the age of 6–18 months), followed by a regression characterized by loss of speech and purposeful hand use and motor apraxia that may be associated with epilepsy and dysautonomic features, including disturbed breathing, sleep and gastrointestinal motility^1,2^. RTT has an incidence of 1:10,000 live female births and is the second cause of intellectual disability after Down’s syndrome in females^3^. RTT was clinically cataloged into classic and atypical forms of the disease. However, these criteria have undergone several updates over the past three decades. Consensus criteria have been established that distinguish RTT patients into the individual classifications of classic or typical RTT and the atypical or variant forms of RTT^4–6^.

A large number of reports support the evidence that mutations in the Methyl CpG binding protein 2 gene (*MECP2*; MIM *300005) are the major causes of classical RTT^7,8^. Over 95% of the cases are explained by more than 800 reported mutations in the methyl CpG-binding protein 2 gene (*MECP2*) (RettBASE: MECP2 Variation Database)^9,10^. MeCP2 is a transcriptional regulatory protein, and in its absence, a large number of genes exhibit abnormal expression with implications in the balance between synaptic excitation and inhibition^11^.

Although the majority of RTT patients have mutations in the *MECP2* gene^10^, approximately 5% of classical RTT and 25% of variant RTT patients are negative for *MECP2* mutation^6,12^. In this variant RTT group of patients, some have mutations in other genes that are also associated with RTT: cyclin-dependent kinase-like5 (*CDKL5*; MIM *300203), which is described in individuals with an early seizure onset variant of RTT^13^, and Forkhead box protein G1 (*FOXG1*; MIM *164874), which is responsible for the congenital variant of RTT^14^. In addition, with the introduction of next generation sequencing (NGS), other genes without previous relation to RTT have been associated with RTT-like phenotypes, such as myocyte-specific enhancer factor 2C (*MEF2C*; MIM *600662) and transcription factor 4 (*TCF4*; *602272)^15^. However, the etiology of a subset of patients with a clinical diagnosis of RTT or RTT-like symptoms remains unknown.

In recent years, NGS has emerged as a potentially powerful tool for the study of this type of genetic disease^16,17^. Now, multiple genes can be sequenced at the same time and at a comparable cost to the Sanger analysis of only one single gene^18,19^. However, for genetic diagnostics, Sanger sequencing still remains necessary to validate the detected variants to avoid false positives.

Here, we present the retrospective results from our group using Sanger sequencing and three different approaches for library construction of NGS libraries. Our main goal was to assess the relative advantages and disadvantages of each methodology for diagnostic purposes.

## Results

A total of 1577 patients with RTT-like syndrome were genetically analyzed between 1999 and 2016 at Sant Joan de Déu Hospital, Barcelona (see Fig. S1). These patients had been diagnosed following the usual clinical parameters^6^ and according to the recently revised RTT Search International Consortium criteria and nomenclature^6^. Throughout the Sanger period, 84% of RTT classical phenotypes and 16% of atypical phenotypes were recruited for study. From 2012, a wider group of patients was recruited, not only RTT classical and atypical but also with RTT-like features, due to the incorporation of the NGS technologies.

Four approaches were used to genetically analyze all RTT patients who were recruited. A total of 1341 patients were studied based on Sanger sequencing (SS). The high throughput approaches were based on three distinct gene library preparations. First, 242 patients were studied using the Haloplex Custom Panel (HCP, Agilent Technologies) (Santa Clara, California), including 46 that came from a negative study of SS. Second, 51 patients were studied using the TruSight One panel (TSO, Illumina) (San Diego, California), including 11 who came from a negative study of HCP. Finally, 22 patients declared as negative-SS and 3 declared as negative-HCP were studied using the SeqCap EZ Human Exome v2.0 and v3.0 (WES, Roche NimbleGen) (Madison, Wisconsin). SS was used to study the exons and surroundings of the three genes associated with RTT (*MECP2*, *CDKL5* and *FOXG1*). HCP is a custom panel of 17 genes designed to cover the exons and surroundings of genes associated with RTT-like phenotypes. TSO targets 4,813 genes associated with a clinical phenotype, including RTT-like phenotypes. The WES kit was used to target all human coding exons, including genes covered by panels.

### Run and Mapping Quality

**Figure 1:**
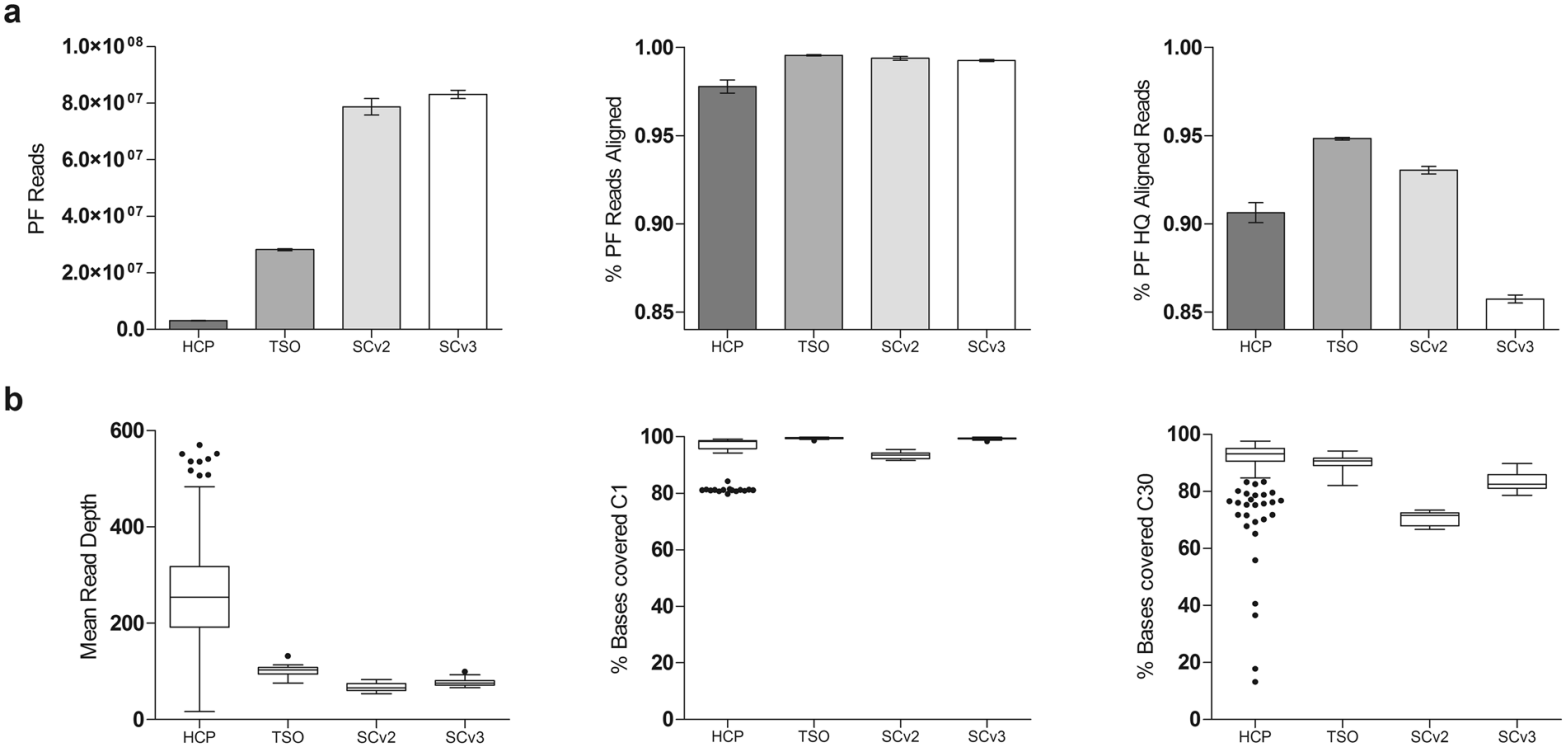
Comparison of main coverage metrics. Average of all samples analyzed for the four different approaches. (**a**) Bar plots with 95% confidence interval for the four approaches. Alignment metrics: passing filter (PF) reads; percentage of PF reads that aligned to the reference sequence; and percentage of PF reads that were aligned to the reference sequence with a mapping quality of Q20 or higher signifying. (**b**) Tukey boxplots: Mean read depth; percentage of bases covered at C1; and percentage of bases covered at C30.

A summary of quality control data is represented in Fig. 1a. The total number of passing filter reads (PF reads) was approximately 9 times higher in TSO (approx. 28 million reads) than HCP (approx. 3 million reads), whereas WES (approx. 80 million reads) was 3 times higher than TSO. The percentage of unique–mapping reads aligned to the reference sequence was higher in TSO (99.5%) and WES (99.4% and 99.2%) than HCP (97.8%). In addition, the unique–mapping reads aligned with a mapping quality Q20 or higher, indicating that the aligner estimates a 1/100 (or smaller) chance of a wrong alignment, was also higher in TSO (94.8%) and WES (93.0 and 85.7%) than HCP (90.6%).

### Target Regions, Read Depth and Coverage

The diagnosis regions of interest for a given set of genes that are related to RTT-like features is defined as the sum of the targets defined by the coding bases of the exons plus a 25 bp flanking region. Three sets of genes of interest were defined where each set included the previous one: RTT list, which included the 3 genes related to RTT; RTT-like list, 3+60 genes related to RTT-like disease; and RTT+EEP list, 3+14 RTT-like genes and 526 genes related to RTT-like features and epileptic encephalopathy (EEP) (Table 1S). Depending on the NGS methods and analyses performed, we evaluated the set of 17 genes common to both panels (TSO and HCP) and the WES, or the set of 605 genes most included in the TSO and all of them in the WES. The gene lists are shown in Supplementary Table 1S.

**Figure 2:**
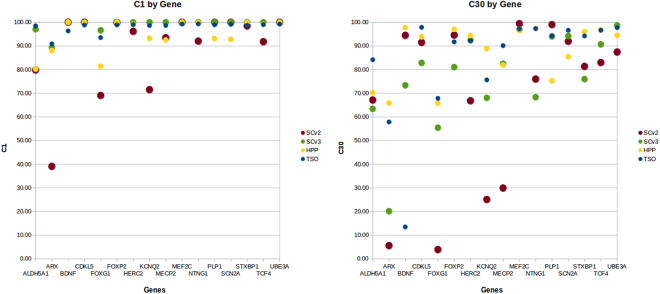
Comparison of coverage in 17 RTT-like genes. Scatter plots of average of the coverage at C1 and C30 of all samples analyzed for the four different approaches.

The performance of the three approaches was compared as if they were four since the WES samples were captured with two different kits, i.e., with SureSelect version 2.0 in 2011 (SCv2) and version 3.0 (SCv3) in 2014. In this study, 30 reads per base was considered the minimum coverage (C30) for high-sensitivity heterozygote detection. For the RTT-like list, average mean read depths of 262×, 99×, 67× and 77× were obtained by HCP, TSO, SCv2 and SCv3, respectively. Ninety percent of the targeted bases were covered at C30 by HCP, and for TSO, 88% of the bases were, while WES was 70% in 2011 and 84% in 2014. These mean coverage results are further compared in Fig. 1b, displaying variation among samples with the four methods referring to the mean read depth and bases covering C1 and C30. Both the coverage and the uniformity of the capture sequencing were better in TSO and WES than HCP. Deepening the coverage data, we analyzed the C1 and C30 of the 16 genes related to an RTT-like phenotype included in HCP considering the four approaches. For the C1 and C30 plots (Fig. 2), we used the raw CCDS coding exon coordinates as common references to compare as fairly as possible the different capture approaches. In this analysis, we discarded the *SHANK3* gene since this gene does not have a CCDS ID (Fig. 2).

### Variant detection

According to diagnostic quality standards, all regions not reaching the required 30× must be Sanger sequenced; from HCP samples, 2 regions of the *MECP2* gene were sequenced by SS. Non-targets were sequenced by SS in TSO and WES. To identify the potential mutations, we checked the variants by matching their affected phenotypes and inheritance patterns of respective genes checked by SS of the index cases and their progenitors. Moreover, we considered the pathogenicity predictors (Mutation Tester, Polyphen-2 and Sorts Intolerant From Tolerant) and reviewed the literature, the RettBASE: MECP2 Variation Database, the Exome Aggregation Consortium (ExAC), HGMD® Professional 2016.4 and The Database of Short Genetic Variation (dbSNP). The potentially pathogenic variants detected in the genes that were not *MECP2*, *CDKL5* or *FOXG1* are shown in Table 1. All results are shown in Supplementary Table S2.

**Table 1:** Potentially pathogenic and causative SNVs detected, excluding RTT genes.

Num. Patients	Gene	OMIM number	Transcript	Type of seq. Change	cDNAchange	Proteinchange	dbSNP	Mutation taster, SIFT, PROVEAN, PolyPhen-2 scores
**Potentially pathogenic mutation detected by HCP**								
2	STXBP1	602926	NM_003165	Missense	c.874C>T	p.Arg292Cys	—	Disease causing, 0, 0.996, −7.58
2	STXBP1	602926	NM_003165	Missense	c.875G>A	p.Arg292His	rs796053361	Disease causing, 0, 1, −4.74
1	KCNQ2	602235	NM_172107	Missense	c.593G>A	p.Arg198Gln	rs796052621	Disease causing, 0, 1, −3.58
1	KCNQ2	602235	NM_172107	Missense	c.637C>T	p.Arg213Trp	rs118192203	Disease causing, 0, 1, −7.19
1	SLC2A1	138140	NM_006516	Missense	c.805C>T	p.Arg269Cys	rs200247956	Disease causing, 0, 1, −7.79
1	STXBP1	602926	NM_003165	Missense	c.1216C>T	p.Arg406Cys	rs796053367	Disease causing, 0, 1, −7.86
1	STXBP1	602926	NM_003165	In-frame deletion	c.124_126delTCC	p.Ser42del	—	Disease causing, NA, NA, −10.71
1	STXBP1	602926	NM_003165	Splicing variant	c.326-3C>G	Miss-splicing	—	NA, NA, NA, NA
1	STXBP1	602926	NM_003165	Missense	c.704G>A	p.Arg235Gln	—	Disease causing, 0, 1, −3.79
1	TCF4	602272	NM_001243236	In-frame indel	c.1169_1175delTAGAAAGinsAAA	p.Leu390Ter	—	Disease causing, NA, NA, NA
1	TCF4	602272	NM_001243236	Missense	c.1733G>A	p.Arg578His	rs121909123	Disease causing, 0, 1, −4.73
1	TCF4	602272	NM_001243236	Nonsense	c.1774C>T	p.Gln592Ter	—	Disease causing, NA, NA, NA
1	TCF4	602272	NM_001243236	Frameshift deletion	c.514_517delAAAG	p.Lys172PhefsTer61	rs398123561	Disease causing, NA, NA, NA
**Potentially pathogenic mutation detected by TSO**								
1	MEF2C	600662	NM_001193347	Missense	c.48C>G	p.Asn16Lys	—	Disease causing, 0.013, 0.995, −5.35
1	MEF2C	600662	NM_001193347	Frameshift deletion	c.989_990delGT	p.Gly330AspfsTer7	—	Disease causing, NA, NA, NA
1	SCN2A	182390	NM_001040142	Missense	c.3631G>A	p.Glu1211Lys	rs387906684	Disease causing, 0, 0.995, −3.82
1	SCN2A	182390	NM_001040142	Missense	c.5317G>A	p.Ala1773Thr	—	Disease causing, 0, 1, −3.68
1	SYNGAP1	603384	NM_006772	Frameshift deletion	c.2019delA	p.Thr674ProfsTer36	—	Disease causing, NA, NA, NA
1	SYNGAP1	603384	NM_006772	Frameshift deletion	c.1782delC	p.Leu595CysfsTer55	rs587780470	Disease causing, NA, NA, NA
**Potentially pathogenic mutation detected by WES**								
1	CACNA1I	608230	NM_021096	Missense	c.4435C>T	p.Leu1479Phe	—	Disease causing, 0.397, 0.756, −1.53
1	CHRNA5	118505	NM_000745	Missense	c.748C>A	p.Pro250Thr	—	Disease causing, 0.301, 1, −6.07
1	GABBR2	607340	NM_005458	Missense	c.1699G>A	p.Ala567Thr	—	Disease causing, 0.002, 0.999, −3.48
1	GRIN2B	138252	NM_000834	Missense	c.1657C>A	p.Pro553Thr	—	Disease causing, 0.001, 0.975, −6.8
1	HCN1	602780	NM_021072	Missense	c.1159G>T	p.Ala387Ser	—	Disease causing, 0.002, 0.767, −2.76

Variant effect predictors web tools used: Mutation taster (http://www.mutationtaster.org/); SIFT-PROVEAN, SIFT scores ranged from 0–1, where 0 is predicted to be most damaging and Protein Variation Effect Analyzer (PROVEAN) score ≤−2.5, the protein variant is predicted to have a “deleterious” effect, while if the PROVEAN score is >−2.5, the variant is predicted to have a “neutral” effect (http://provean.jcvi.org/genome_submit_2.php); and Polyphen-2, ranged from 0–1, where 1 is most likely to be damaging (http://genetics.bwh.harvard.edu/pph2/).

### Sanger Sequencing-SS

A total of 1341 patients were studied by SS between 1999 and 2012, and 375 (22 by MLPA) were genetically diagnosed (28%) with mutations in *MECP2*, *CDKL5* or *FOXG1*. During this period of time, the workflow was to study the four exons of the *MECP2* gene to detect SNVs or short indels and MLPA for gross rearrangements. Excluding large rearrangements detected by MLPA, a total of 293 patients with RTT classic and 36 with atypical phenotypes had mutations in *MECP2*. Then, the *CDKL5* and *FOXG1* genes were studied in the patients without mutations in *MECP2*. No patient diagnosed with RTT-classic had mutations in *CDKL5* or *FOXG1*, and 15 patients with atypical RTT had mutations in *CDKL5* and 9 in the *FOXG1* gene.

### Haloplex Custom Panel - HCP

A total of 242 patients were studied with HCP between 2012 and 2016, and 53 (6 by MLPA) patients were genetically diagnosed (22%). Excluding large rearrangements detected by MLPA, 29 patients had mutations in RTT genes and 90% in *MECP2*. From 18 patients with mutations in RTT-like genes, it is remarkable that the majority of these mutations were in the *STXBP1* gene, which is associated with early infantile epileptic encephalopathy (EIEE4)^20^, and the *TCF4* gene, which is associated with Pitt–Hopkins syndrome^21^.

### TruSight One panel - TSO

Fifty-one patients were studied by TSO since 2015, and 15 patients were genetically diagnosed (29%). Three patients had mutations in RTT genes and 12 in RTT-like genes. In these 12 patients, we detected 2 SNVs in *MEF2C* and 2 in *SCN2A* genes, which are associated with mental retardation, stereotypic movements, epilepsy, and/or cerebral malformations^22–24^; 2 SNVs in the *SYNGAP1* gene, which is associated with mental retardation^24,25^; a deletion of *IQSEC2* and *KDM5C* genes, which are associated with mental retardation^26^; a gross deletion in chr15 (chr15:22,833,395-28,567,298), which is associated with Prader-Willi syndrome^27^; and a duplication in chr14 (14q32.11-q32.33(90949120-107287505)), which is associated with mental retardation and development delay^28^. All CNVs detected through the read depth and detected by NGS were confirmed by CGHarray.

### *Whole Exome Sequencing* - WES

Twenty-five patients were studied by WES in 2011 (SCv2) and 2014 (SCv3). Five occurred in genes previously associated with neurodevelopmental disorders with features similar to those of RTT syndrome. We identified four mutations in genes such as *HCN1*^29^ and *GRIN2B*^30^, which are associated with early infantile epileptic encephalopathy; *SLC6A1*, which is associated with epilepsy and myoclonic-atonic seizures^31^; *TCF4*, which is associated with Pitt–Hopkins syndrome^21^; and *SCN1A*, which is associated with Dravet syndrome^32^. The other potentially pathogenic variants that were identified occurred in genes that had not been linked to any genetic disorder^33^. However, there was an enrichment of genes with a potential role in neuronal biology and functionality, such as the gamma-aminobutyric type B receptor subunit 2 (*GABBR2*), the neuronal acetylcholine receptor subunit alpha-5 (*CHRNA5*), and the neuronal voltage-gated calcium channel (*CACNA1I*).

### Genetic diagnosis

**Figure 3:**
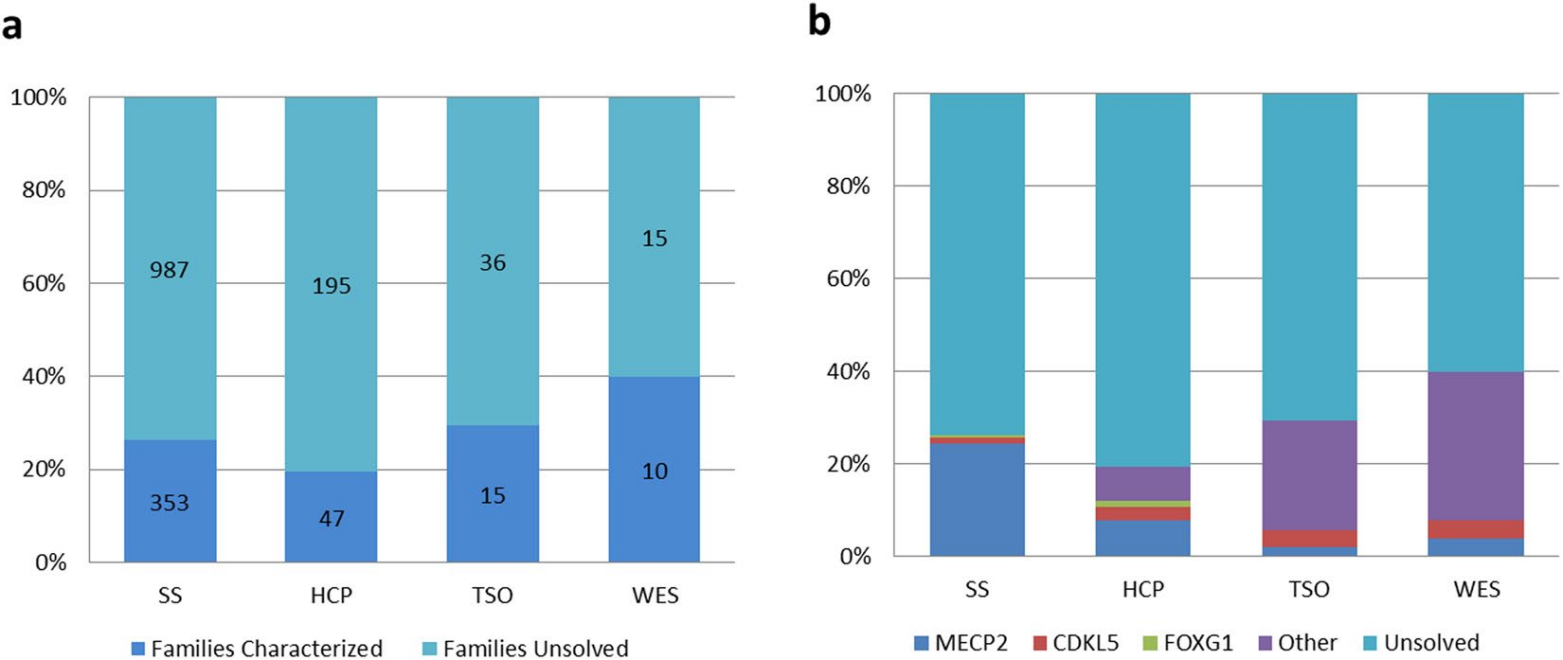
Statistics results for genetic diagnosis. Each columns represents different detection methodology used (SS, HCP, TSO and WES) (**a**) Percentage and total of families characterized and unsolved by the four different approaches. (**b**) Percentage of patients with *MECP2*, *CDKL5*, *FOXG1*, other genes with pathogenic mutations and the unsolved cases for the Sanger Sequencing (SS) and the three NGS approaches. Total of mutations found are detailed in Table S2.

The total of cases and statistical results of genetic diagnoses of the RTT-like patients are listed in Fig. 3a. We identified the genetic cause for 353/1341 patients studied with SS. All characterized patients (26.3%) had mutations in RTT genes (*MECP2*, 24.5%; *CDKL5*, 1.1%; *FOXG1*, 0.7%). Forty-seven of 242 patients (19.4%) were genetically diagnosed by HCP; for these patients, 7.9% had mutations in the *MECP2* gene, 2.9% in *CDKL5*, 1.2% in *FOXG1* and 7.4% in other genes related to RTT-like phenotypes. Fifteen of 51 patients (29.4%) were genetically diagnosed by TSO; for these patients, 2.0% had mutations in the *MECP2* gene, 3.9% in *CDKL5* and 23.5% in other genes related to RTT-like phenotypes. Ten of 25 patients (40.0%) were genetically diagnosed by TSO; for these patients, 4.0% had mutations in the *MECP2* gene, 4.0% in *CDKL5* and 32.0% in other genes related to RTT-like phenotypes (Fig. 3b).

In addition of these results, it has been identified by MLPA twenty four patients with gross deletions which are not included in the statistical results. Twenty one patients with gross deletions in MECP2, where one have a deletion of exon 1 and 2, six patients in exon 3 and fourteen patients in exon 3 and 4. Two patients have a deletion in *CDKL5*, one with a deletion in exon 1 and 2 and other in exon 8. And three patients with a deletion FOXG1.

## Discussion

Over the last few years, several genes have been associated with RTT-like phenotypes^17,33–37^ due to the incorporation of NGS. Traditional detection methods for individual genes such as SS can only provide a limited mutation spectrum of the disease, consuming a great amount of time. NGS, which has revolutionized molecular genetics research, is a high-throughput method capable of rapidly sequencing a large number of genes in parallel and providing large datasets^33–38^. This study reports a comparison among different sequencing methods used across the last decade. We first developed a custom panel for molecular diagnosis of RTT and RTT-like phenotypes covering 17 genes related to RTT-like disease and then used a commercial panel that includes these genes and others also associated with the phenotype. Furthermore, we performed WES to identify unsolved families via SS or a custom panel.

High performance standards are essential in a clinical diagnostic setting. Even though the mean read depth in HCP is ostensibly higher than other methods, with regions covering more than 500×, the uniformity of the capture is uneven. Figure 1b shows that some samples from different captures are in the border or below the quality standards of diagnosis. In spite of the fact that TSO has a slightly lower coverage at C30 than HCP, the homogeneity and uniformity of all the samples analyzed is more accurate. Even though the number of target region bases not reaching C30 is much higher in WES than in panel sequencing, it should be noted that the quality of the analysis improved in these few years (2011 to 2014). Moreover, these coverage comparatives could be inequitable, and we have to take into account that these WES are not performed with very high coverage and we cannot eliminate duplicates of PCR of the HCP. Thus, we not only compared the C30 but also the C1, which is the region that could potentially be covered if adequate coverage was made. Comparing the C1, TSO and SCv3 could potentially be better coverage than HCP and SCv2^39^.

A large cohort of Spanish patients who exhibited clinical features associated with RTT or RTT-like phenotypes was recruited over three decades. Before the implementation of NGS, the cohort of patients was less clinically homogenous. Today, the cohort presents an increase in the number of patients who were RTT-like compared to classic or atypical RTT. This is likely due to the limitation of the technique because it takes time to analyze the 3 genes associated with RTT using SS and clinicians only send samples from patients who have fulfilled the clinical criteria as RTT.

Regarding additional information of this study, with a more extensive panel, the rate of the families characterized was higher. We characterized 40% of the families analyzed by WES and only 26% by SS. It is obviously less costly to sequence a panel of patients than an exome per trio (patient and progenitors) due to the much smaller capture region. Moreover, the development of panels targeting most clinically relevant genes, such as TSO or HCP, would be more cost-effective than standard WES. However, it must be taken into consideration that our HCP panel required a cost for development and validation, and TSO and WES are both labelled for research use only. For these reasons, proper validation by the laboratory is required before implementation in patient diagnoses. On the other hand, data obtained in a HCP or TSO are easier to interpret than WES results and also the computational requirements for a panel of a few genes is lower than a WES^40^.

Regarding the NGS results, the *MECP2* gene remains the major mutated gene in our cohort (82% of all positive results), followed by *CDKL5* (6%) and *FOXG1* (3%) genes. One of the important findings is that two genes related to other phenotypes were found to be more frequently mutated than in other RTT-like phenotypes. Eight patients had pathogenic mutations in the *STXBP1* gene. The clinical features in patients with a mutation in this gene, such as developmental regression in the neonatal period or infancy, hypotonia, poor visual pursuit, seizures and epileptic encephalopathy, could fit perfectly with the RTT phenotype. Five patients had pathogenic mutations in the *TCF4* gene and one in the *UBE3A* gene, which are associated with Pitt-Hopkins syndrome and Angelman syndrome, respectively. Mutations in these genes cause severe neurologic features such as poor or absent speech development, delayed motor development, seizures, and hypotonia with an onset during the first year of life. Moreover, the patients with Pitt-Hopkins have morphologic characteristics, such as deep-set eyes and fleshy ears, but in our cohort, we found patients without any of these features. We also found 2 patients with mutations in the *MEF2C* gene (mental retardation, stereotypic movements, epilepsy, and/or cerebral malformations) and 2 patients with mutations in the *SYNGAP1* gene (mental retardation, autosomal dominant 5). The fact that there are the same neurologic features commented on previously highlights that *SYNGAP1* has behavioral psychiatric manifestations identical to RTT, that is, autism features and regression of motor development.

It is also remarkable that, in addition to *STXBP1* (EEIE4), we found mutations in genes related to EEIE: *KCNQ2* (EEIE7), *SCN1A* (EEIE6)*, SCN2A* (EEIE11), *GRIN2B* (EEIE27) and *HCN1* (EEIE24) genes. Furthermore, we also found members of the solute carrier families related to epilepsy: *SLC6A1* (myoclonic-atonic epilepsy) and *SLC2A1* (idiopathic generalized epilepsy) genes^33,35^.

Finally, mutations in genes without related phenotypes were detected by WES: *GABBR2, CHRNA5* and *CACNA1I*. Even though these genes are quite unknown, they have a potential role in neuronal biology and functionality. Further functional studies are required to consider these as the genetic cause of the disease. The clinical characteristics and detected variants of these patients are summarized in Lucariello *et al*.^33^.

In this study, we focused on the comparison of a single nucleotide variant (SNV) and insertion/deletion detection, since these approaches are highly sensitive and specific to detect these types of mutations. Therefore, our diagnostics workflow includes MLPA (Multiplex Ligation-dependent Probe Amplification) of the *MECP2*, *CDKL5*, *FOXG1* and *TCF4* genes. aCGH (array comparative genomic hybridization) can be used for patients who have to be analyzed by WES. Although copy number variations (CNV) are difficult to detect with these NGS methods, we performed a preliminary study with TSO read depths of patients without detecting SNVs, trying to detect CNVs via bioinformatics methods. We were able to detect 3 *de novo* pathogenic CNVs corroborated later by aCGH: two deletions, one of the *IQSEC2* gene and one of the *KDM5C* gene, and another in chr15:22,833,395-28,567,298. One gross duplication in the long arm of chr14:90,949,120-107,287,505 was found.

In summary, the genetic study by NGS allows study of a larger number of genes associated with RTT-like symptoms simultaneously, allowing genetic study of a wider group of patients^10,41^. These detected variants identified by NGS may modify the initial clinical diagnosis to other neurodevelopmental syndromes, or determine new candidate genes related to RTT-like symptoms, providing the clinician with more information and clues that could help in the prevention of future symptoms or in the pharmacologic therapy. For instance, the use of D-serine as a dietary supplement for the enhancement of glutamatergic neurotransmission and/or excitatory/inhibitory neurotransmitter imbalance affected in patients with mutations in *N*-Methyl-D-aspartate receptors such as *GRIN2B* gene^42^.We could conclude that TSO has the best cost-efficiency of all technologies used and could offer timely responses for clinical diagnosis. However, performing a WES in families to characterize the family and identify new candidate genes should be included in the target panels. In addition, study of the progenitors remains essential for their characterization as well as the need for functional studies in newly discovered genetic variants.

## Material and Methods

### Patients and DNA extraction

A cohort of 1577 Spanish patients who exhibited clinical features associated with RTT or RTT-like phenotypes was recruited at Sant Joan de Déu Hospital in Barcelona, Spain, from different Spanish Hospitals. Genomic DNA samples were extracted from peripheral blood leukocytes using the Puregene DNA Isolation kit (Gentra System, Minneapolis, USA) following the manufacturer’s instructions. All DNA samples were quantified using a Qubit 2.0 Fluorometer (Invitrogen), and the DNA purity was quantified by calculating the absorbance ratio (*A*260/280) with a NanoDrop 1000 (Thermo Fisher).

### Ethical issues

The study was approved by the ethical committees of Hospital Sant Joan de Déu, CEIC: Comité d’Ètica d’Investigación Clinica- Fundació Sant Joan de Déu (internal code: PIC-101-15). Patients or their parents signed informed consent for genetic studies, and tissue samples from patients and controls were obtained according to the Helsinki Declaration of 1964, as revised in 2001.

### Molecular analysis

A total of 1341 patients were sequenced by SS for the 3 RTT genes (*MECP2*, *CDKL5* and *FOXG1*); 242 patients by HCP; 51 patients by TSO; and 24 patients with their healthy progenitors by WES. All patients analyzed by WES had an aCGH performed first with a normal or inconclusive profile.

### Copy Number Variations - CNVs

#### Multiplex Ligation-dependent Probe Amplification (MLPA)

*MECP2*-MLPA was performed using a SALSA kit P015, *CDKL5-*MLPA with a SALSA kit P189 and *FOXG1-TCF4-*MLPA with a SALSA kit P075 (MRC-Holland, Amsterdam, The Netherlands) according to the manufacturer’s instructions.

#### Array Comparative Genomic Hybridization (aCGH)

The aCGH analysis was performed at Bioarray (Genetic diagnosis, Alicante, Spain) using two different platforms: human genome Cytoarray Plus 180K and 400K (Agilent Technologies, Santa Clara, CA, USA). All genomic coordinates are in build GRCh37/hg19.

### NGS: Library Preparation

Library preparation was conducted according to the manufacturers’ instructions. HCP and TSO panels were created at Sant Joan de Déu Hospital and WES at the National Center for Genomic Analysis (CNAG).

#### Haloplex Custom Panel (HCP)

We designed a custom-made panel with 17 genes associated with a RTT-like phenotype based on the evidence curated in the Online Mendelian Inheritance in Man (OMIM). The genes are shown in Supplementary Table S3. Amplicon libraries were prepared using the Agilent HaloPlex Target enrichment system, for Illumina paired-end multiplexed sequencing platforms (Agilent Technologies), according to the manufacturer’s sample preparation protocol. Briefly, 225 ng of genomic DNA was digested with restriction enzymes. The hybridization was performed for 3 hours at 54 °C, and the circularized target DNA-HaloPlex probe hybrids, containing biotin, were captured on streptavidin beads (HaloPlex Magnetics Beads, Agilent Technologies). The DNA with adaptor-modified ends was PCR amplified (number of cycles depended on the lot, Herculase II fusion DNA polymerase, Agilent). The amplified target DNA was purified using AMPure XP beads (Beckman Coulter Genomics, GENEWIZ, New Jersey, USA). All of the DNA samples were individually indexed. Amplification of the libraries was performed on a GeneAmp®PCR System 9700 thermocycler (Applied Biosystems). The restriction digestion and amplicon library quantities were quality evaluated using a Bioanalyzer High Sensitivity DNA Assay kit in an Agilent 2100 Bioanalyzer (Agilent Technologies) and quantified using a Qubit 2.0 Fluorometer (Invitrogen).

#### TruSight One Panel (TSO)

TruSight One Sequencing Panel (Illumina, San Diego, CA) targeted 4,813 genes associated with a clinical phenotype. Libraries were generated using the TruSight One^TM^ Sequencing Panel kit with the TruSight One^TM^ Sequencing Panel (Illumina), according to the manufacturer’s sample preparation protocol. Briefly, 50 ng of each DNA sample was enzymatically fragmented and adapter sequences were added to the ends. The fragmented DNA was purified, and barcodes and common adapters required for cluster generation and sequencing were PCR-added. After cleanup, 500 ng of each of the 12 DNA libraries was pooled. Then the libraries were hybridized twice to specific capture probes; the unhybridized material was washed away, and the captured fragments were amplified using PCR followed by purification. The enriched libraries were quantified using a Qubit 2.0 Fluorometer, and their quality was evaluated using a Bioanalyzer 2100 and the High Sensitivity DNA Kit (Agilent Technologies). Libraries were diluted and pooled to obtain the final sequencing equimolar pool.

#### Whole Exome Sequencing (WES)

For whole exomes from 2011, the sample preparation for capturing approximately 44 Mb of selected human genome regions was performed according to the NimbleGen SeqCap EZ Exome Libray SR protocol, v2.2, for Illumina paired-end sample libraries with modifications included in draft v1.4 (February 2011). In brief, 1.0 μg of genomic DNA was sheared on a Covaris™ E210 instrument (Covaris). The fragment size (150–400 bp) and the quantity were confirmed with the Agilent 2100 Bioanalyzer 1000 chip (Agilent). Fragmented DNA was prepared using an Illumina TruSeq DNA Sample Preparation Kit (Illumina) following the protocol described in the Illumina TruSeq DNA Sample Preparation Guide (revision A, November 2010) with the exception of the fragment size selection using an Agencourt SPRI XP Kit (Beckman Coulter). Instead of the standard Illumina PCR enrichment step, amplification via pre-capture LM-PCR (8 cycles) was performed with a SeqCap EZ Human Exome Kit v2.0 (Roche NimbleGen). One microgram of the amplified library was hybridized to EZ probes at 47 °C for 72 hrs. After washing and recovery of the captured DNA, the library was amplified through post-capture LM-PCR (18 cycles). The final product was quality controlled on a Bioanalyzer DNA 1000 chip, and the success of the enrichment was measured with a qPCR SYBR Green assay on a LightCycler® 480 Instrument (Roche), evaluating one genomic locus with pre- and post-captured material.

For whole exomes from 2014, the NimbleGen SeqCap EZ v3.0 system for exome enrichment was used and pre-capture multiplexing was applied following the manufacturer's protocol version 4.2. Briefly, 1 μg of genomic DNA was fragmented with Covaris ™E210 and used for ligation of the adapters containing Illumina specific indexes with a KAPA Library Preparation kit (Kapa Biosystems). Adapter ligation DNA fragments were enriched by 7 cycles of pre-capture PCR using KAPA HiFi HotStart ReadyMix (2×) (Kapa Biosystems) and analyzed on an Agilent 2100 Bioanalyzer with the DNA 1000 assay. Five libraries were pooled with a combined mass of 1250 ng for the baits hybridization step (47 °C; 68 hrs). After washing (47 °C), the multiplexed captured library was recovered with capture beads and amplified with 14 cycles of post-capture PCR using KAPA HiFi HotStart ReadyMix (2×). The size, concentration and quality of the captured library were determined using an Agilent DNA 1000 chip. The success of the enrichment was measured using a qPCR SYBR Green assay on a Roche LightCycler® 480 Instrument evaluating one genomic locus with pre- and post-captured material.

### NGS: Run

HPC and TSO libraries were sequenced on an Illumina Miseq instrument and NextSeq 500, respectively, following the manufacturer’s protocol, with a paired end run. Each WES (2011 and 2014) library was sequenced on an Illumina HiSeq 2000 instrument in a fraction of a sequencing lane following the manufacturer’s protocol, with a paired end run of 2×101 bp. Image analysis, base calling and quality scoring of the run were processed using the manufacturer’s software Real Time Analysis (RTA 1.10.36 and RTA 1.13.48) and followed by generation of FASTQ sequence files by CASAVA.

### NGS: Data Analysis

HPC and TSO data analyses were performed at Genycell Biotech S.L. (Madrid, Spain). Briefly, both panel sequencing reads were trimmed from the 3′ end up to the first base with a Phred quality >9 and were mapped to the Human genome reference v37 with decoy sequences [hs37d5] (Broad, ftp://ftp.1000genomes.ebi.ac.uk/vol1/ftp/technical/reference/phase2_reference_assembly_sequence/hs37d5.fa.gz) using the BWA-MEM version 0.7.5a. (bio-bwa.sourceforge.net/). To calculate read statistics, we used Prinseq-lite version 0.20.3 lite (prinseq.sourceforge.net/) and Picard Calculate HS Metrics version 1.119 (http://picard.sourceforge.net) to calculate metrics. Alignment (.bam) files containing only properly paired and uniquely mapped reads were processed with Picard Mark Duplicates version 1.119 to remove duplicates, and local realignment was performed with Freebayes version 9.9.13 (https://github.com/ekg/freebayes)^43^. SAMtools version 0.1.19 (http://samtools.sourceforge.net/)^44^ was used on the processed BAM files to call single nucleotide variants (SNVs) and small insertion deletions (INDELs). Functional annotations from Ensembl release 75 (GRCh37.75 database)^45^ were added to the resulting.vcf files using the Genome Analysis Tool Kit (GATK) version 2.4 (https://www.broadinstitute.org/gatk/)^46^ to annotate variants with dbSNP version 137 and Ensembl Variant Effect Predictor version 72 (http://www.ensembl.org/info/docs/tools/vep/index.html) to annotate the variants.

For each WES (2011 and 2014), sequencing reads were trimmed from the 3′ end up to the first base with a Phred quality >9 and were mapped to the Human genome reference v37 with decoy sequences [hs37d5] using the GEM toolkit^47^. Alignment (.bam) files containing only properly paired and uniquely mapped reads were processed with Picard tools version 1.110 (http://picard.sourceforge.net) to remove duplicates, and local realignment was performed with the Genome Analysis Tool Kit (GATK) version 3.1 (https://www.broadinstitute.org/gatk/)^46^. For alignments and coverage metrics, it has been remapped with BWA-MEM version 0.7.5a^48^. To call single nucleotide variants (SNVs) and small insertion deletions (INDELs) on the processed alignment (.bam) file, we used SAMtools version 0.1.19 (http://samtools.sourceforge.net/)^44^. Functional annotations from Ensembl release 75 were added to the resulting VCF using snpEff (http://snpeff.sourceforge.net/)^49^. SNPSift (http://snpeff.sourceforge.net/ SnpSift.html)^50^ was used to add information from dbSNP version 137, population frequencies from 1000 Genomes and the Exome Variant Server^51^, the NHLBI Exome Sequencing Project (http://evs.gs.washington.edu/EVS/) and a variety of conservation and deleteriousness predictions included in dbNSFP version 2.5 (http://sites.google.com/site/jpopgen/dbNSFP)^52^.

### Sanger validation

The identified variants and familial segregation studies were validated by SS. The specific primers were designed online by Primer3 version 0.4.0 (http://bioinfo.ut.ee/primer3-0.4.0/primer3/). The PCR products were sequenced using a Big-Dye® Terminator version 3.1 Cycle Sequencing Kit in an Applied Biosystems 3730/DNA Analyzer (Applied BioSystems, Waltham, Massachusetts, USA). The raw data were analyzed with Chromas trace viewer (http://technelysium.com.au/wp/chromas/). The primers used for SS are shown in Supplementary Table S4.

## Electronic supplementary material

Supplementary files
