# Author Correction: The utility of Next Generation Sequencing for molecular diagnostics in Rett syndrome

**DOI:** 10.1038/s41598-021-97262-y

**Published:** 2021-09-07

**Authors:** Silvia Vidal, Núria Brandi, Paola Pacheco, Edgar Gerotina, Laura Blasco, Jean-Rémi Trotta, Sophia Derdak, Maria del Mar O’Callaghan, Àngels Garcia-Cazorla, Mercè Pineda, Judith Armstrong, Rett Working Group, Francisco Javier Aguirre, Montserrat Aleu, Xènia Alonso, Mercè Alsius, Maria Inmaculada Amorós, Guillermo Antiñolo, Lourdes Aquino, Carmen Arellano, Gema Arriola, Rosa Arteaga, Neus Baena, Montserrat Barcos, Nuria Belzunces, Susana Boronat, Tomás Camacho, Jaume Campistol, Miguel del Campo, Andrea Campo, Ramon Cancho, Ramon Candau, Ignacio Canós, María del Carmen Carrascosa, Francisco Carratalá-Marco, Jovaní Casano, Pedro Castro, Ana Cobo, Jaime Colomer, David Conejo, Maria José Corrales, Rocío Cortés, Gabriel Cruz, Gábor Csányi, María Teresa de Santos, María de Toledo, Mireia Del Toro, Rosario Domingo, Anna Duat, Rosario Duque, Ana María Esparza, Rosa Fernández, Maria Carme Fons, Ana Fontalba, Enrique Galán, Pia Gallano, María José Gamundi, Pedro Luis García, María del Mar García, María García-Barcina, María Jesús Garcia-Catalan, Sixto García-Miñaur, Juan Jose Garcia-Peñas, María Teresa García-Silva, Rosa Gassio, Esther Geán, Belén Gil, Sarenur Gökben, Luis Gonzalez, Veronica Gonzalez, Julieta Gonzalez, Gloria González, Encarna Guillén, Miriam Guitart, Montserrat Guitet, Juan Manuel Gutierrez, Eva Gutiérrez, Jose Luís Herranz, Gemma Iglesias, Iva Karacic, Carlos H. Lahoz, José Ignacio Lao, Pablo Lapunzina, María Jesús Lautre-Ecenarro, María Dolores Lluch, Laura López, Asunción López-Ariztegui, Alfons Macaya, Rosario Marín, Charles M. Lourenço Marquez, Elena Martín, Beatriz Martínez, Eduardo Martínez-Salcedo, María José Mas, Gonzalo Mateo, Pilar Mendez, Amparo Morant Jimenez, Sira Moreno, Fernando Mulas, Juan Narbona, Andrés Nascimento, Manuel Nieto, Tania Fabiola Nunes, Núria Núñez, María Obón, Ignacio Onsurbe, Carlos Ignacio Ortez, Emilio Orts, Francisco Martinez, Rafael Parrilla, Samuel Ignacio Pascual, Ana Patiño, Maria Pérez-Poyato, Belén Pérez-Dueñas, Pilar Póo, Eliodoro Puche, Feliciano Ramos, Miquel Raspall, Ana Roche, Susana Roldan, Jordi Rosell, Cesar Ruiz, María Luz Ruiz-Falcó, Maria Eugenia Russi, Jordi Samarra, Victoria San Antonio, Ivan Sanchez, Xavier Sanmartin, Ana Sans, Alfredo Santacana, Sabine Scholl-Bürgi, Nuria Serrano, Mercedes Serrano, Pilar Martin-Tamayo, Adrián Tendero, Jaime Torrents, Diego Tortosa, Emma Triviño, Ledia Troncoso, Eulalia Turrón, Pilar Vázquez, Carlos Vázquez, Ramón Velázquez, Clara Ventura, Alfonso Verdú, Anna Vernet, M. Tomás Vila, Cristina Villar

**Affiliations:** 1 Molecular and Genetics Medicine Section, Hospital Sant Joan de Déu, Barcelona, Spain; 2 Facultat de Medicina, Universitat de Barcelona, Barcelona, Spain; 3 Centro Nacional de Análisis Genómica (CNAG-CRG), Center for Genomic Regulation, Barcelona Institute of Science and Technology (BIST), Barcelona, Spain; 4 Neurology Service, Hospital Sant Joan de Déu, Barcelona, Spain; 5 Institut de Recerca, Pediàtrica Hospital Sant Joan de Déu, Barcelona, Spain; 6 CIBER-ER (Biomedical Network Research Center for Rare Diseases), Instituto de Salud Carlos III, Madrid, Spain; 7 Hospital Torrecardenas, Almeria, Spain; 8 Consorcio Hospital General Universitario de Valencia, Valencia, Spain; 9 Hospital Universitari Dr. Josep Trueta, Girona, Spain; 10 Hospital Punta Europa, Cadiz, Spain; 11 Hospital Universitario Virgen del Rocio, Sevilla, Spain; 12 Hospital de Mataró, Mataro, Spain; 13 Consorci Sanitari, Terrassa, Spain; 14 Hospital universitario de Guadalajara, Guadalajara, Spain; 15 Hospital Universitario Marqués de Valdecilla, Santander, Spain; 16 Hospital de Sabadell, Barcelona, Spain; 17 Hospital Universitario Reina Sofía, Cordoba, Spain; 18 Balagué Center, Barcelona, Spain; 19 Hospital de la Vall d’Hebrón, Barcelona, Spain; 20 Lema & Bandin Laboratorios, Vigo, Spain; 21 Hospital Universitario Río Hortega, Valladolid, Spain; 22 Hospital Universitario Doctor Peset, Valencia, Spain; 23 Hospital General Universitario de Albacete, Albacete, Spain; 24 Hospital Universitari San Juan, Alicante, Spain; 25 Hospital Universitario General de Castellón, Castellon de la Plana, Spain; 26 Hospital Gregorio Marañón, Madrid, Spain; 27 Hospital de Donostia, San Sebastian, Spain; 28 Complejo asistencial, Burgos, Spain; 29 Hospital General Mancha Centro, Ciudad Real, Spain; 30 Hospital San Borja Arriaran, Santiago, Chile; 31 Hospital Universitario de Valme, Sevilla, Spain; 32 Hospital de la Santa Creu i Sant Pau, Barcelona, Spain; 33 Hospital de Fuenlabrada, Madrid, Spain; 34 Hospital Universitario Severo Ochoa, Madrid, Spain; 35 Hospital Infantil de La Arrixaca, Murcia, Spain; 36 Hospital Infantil Universitario Niño Jesús, Madrid, Spain; 37 Hospital Universitario Nuestra Señora de la Candelaria, Santa Cruz de Tenerife, Spain; 38 Hospital Universitario de Getafe, Madrid, Spain; 39 Hospital Materno-Infantil de Badajoz, Badajoz, Spain; 40 Hospital Virgen de la Salud, Toledo, Spain; 41 Hospital Cormarcal de Figueres, Girona, Spain; 42 Hospital de Basurto, Bilbao, Spain; 43 Hospital La Paz, Madrid, Spain; 44 Hospital Universitario 12 de Octubre, Madrid, Spain; 45 Ege Ünŭversŭtesŭ Tip Fakültesŭ Pedŭatrŭ AD, Uzmur, Turkey; 46 Hospital General de Granollers, Barcelona, Spain; 47 Hospital Clínico Universitario de Valladolid, Valladolid, Spain; 48 Hospital Virgen de la Luz, Cuenca, Spain; 49 Clinical Hospital Center Zagreb, Zagreb, Croatia; 50 Hospital Central Asturias, Asturias, Spain; 51 Laboratorio Echevarne, Barcelona, Spain; 52 Hospital Clínico San Carlos, Madrid, Spain; 53 Hospital Universitario Virgen Macarena, Sevilla, Spain; 54 Hospital de Cruces, Bilbao, Spain; 55 Hospital Universitario Puerta del Mar, Cadiz, Spain; 56 Medical Genetics Service, Clinics Hospital of Ribeirão Preto, University of São Paulo, Sao Paulo, Brazil; 57 Hospital General Universitario de Alicante, Alicante, Spain; 58 Hospital Joan XXIII, Tarragona, Spain; 59 Centro privado, Valencia, Spain; 60 Hospital Virgen del Camino, Pamplona, Spain; 61 Instituto Valenciano de Neurociencias, Valencia, Spain; 62 Clínica Universitaria de Pamplona, Pamplona, Spain; 63 Complejo Hospitalario de Jaén, Jaen, Spain; 64 Hospital de Navarra, Navarra, Spain; 65 Hospital Clínico Universitario Lozano Blesa, Zaragoza, Spain; 66 Hospital Universitario Virgen de las Nieves, Granada, Spain; 67 Hospital Son Dureta, Palma de Mallorca, Spain; 68 Hospital Costa del Sol, Malaga, Spain; 69 Hospital General de Vic, Barcelona, Spain; 70 Complejo Hospitalario Universitario Insular, Las Palmas de Gran Canaria, Spain; 71 Medizinische Universität Innsbruck, Innsbruck, Austria; 72 Althaia, Manresa, Spain; 73 Reference Laboratory, Barcelona, Spain; 74 Catlab, Barcelona, Spain; 75 Hospital Francesc De Borja, Valencia, Spain

Correction to: *Scientific Reports* 10.1038/s41598-017-11620-3, published online 25 September 2017

This Article contains an error in the Acknowledgements section.

“We thank all patients and their families who contribute to this study. The work was supported by grants from the Spanish Ministry of Health (Instituto de Salud Carlos III/FEDER, PI15/01013); Crowdfunding program PRECIPITA, from the Spanish Ministry of Health (Fundación Española para la Ciencia y la Tecnología); Catalan Association for Rett Syndrome; Fondobiorett and Mi Princesa Rett.”

should read:

“We thank all patients and their families who contribute to this study. The work was supported by grants from the Spanish Ministry of Health (Instituto de Salud Carlos III/FEDER, PI15/01159); Crowdfunding program PRECIPITA, from the Spanish Ministry of Health (Fundación Española para la Ciencia y la Tecnología); Catalan Association for Rett Syndrome; Fondobiorett and Mi Princesa Rett.”

