# Supplementary material for: The utility of Next Generation Sequencing for molecular diagnostics in Rett syndrome
Source: Sci Rep. 2017 Sep 25;7:12288. doi: 10.1038/s41598-017-11620-3 (PMC5613000; doi:10.1038/s41598-017-11620-3)

The utility of Next Generation Sequencing for molecular diagnostics in Rett syndrome

Silvia Vidal[1], Núria Brandi[2], Paola Pacheco[1], Edgar Gerotina[1], Laura Blasco[1] Jean-Rémi Trotta[3]; Sophia Derdak[3], Maria del Mar O'Callaghan[4,5,6], Àngels Garcia-Cazorla[4,5,6], Mercè Pineda[5], Judith Armstrong[1,5,6 *] & Rett Working Group

[1]Molecular and Genetics Medicine Section, Hospital Sant Joan de Déu, Barcelona, Spain
[2]Facultat de Medicina, Universitat de Barcelona, Barcelona, Spain,
[3]Centro Nacional de Análisis Genómica (CNAG-CRG), Center for Genomic Regulation, Barcelona Institute of Science and Technology (BIST), Barcelona, Spain
[4]Neurology Service, Hospital Sant Joan de Déu, Barcelona, Spain
[5]Institut de Recerca Pediàtrica Hospital Sant Joan de Déu, Barcelona, Spain
[6]CIBER-ER (Biomedical Network Research Center for Rare Diseases), Instituto de Salud Carlos III, Madrid, Spain

***Corresponding author:**

J. Armstrong ,
Molecular and Genetics Medicine Section
Hospital Sant Joan de Déu
Pg. Sant Joan de Déu 2, planta 0
08950 Esplugues de Llobregat, Barcelona
T. + 34 93 600 9451
F. + 34 93 600 9760
jarmstrong@sjdhospitalbarcelona.org

Table S1. Gene list. Gene list used for filtering in HCP and TSO. Two list have been used: Rett-like gene list, and Epileptic encephalopathy gene list.

**Rett-like gene list**	**Epileptic encephalopathy gene list**
AKT3	*ABAT*
ARHGEF9	*ABCB1*
ARX	*ABCC2*
CACNA1I	*ABCC8*
CACNB4	*ACOX1*
CDKL5	*ACY1*
CHD2	*ADCK3*
CHKB	*GPR56*
CHRNA5	*GPR98*
FOXG1	*ADSL*
GABBR2	*AGA*
GABRA1	*AGTR2*
GABRD	*AHI1*
GABRG2	*AKT3*
GRIN2A	*ALDH4A1*
GRIN2B	*ALDH5A1*
HCN1	*ALDH7A1*
HNRNPU	*ALG1*
KCNQ2	*UTP14C*
KCNQ3	*ALG12*
KCNT1	*ALG13*
MBD5	*ALG2*
MECP2	*ALG3*
MEF2C	*ALG6*
PCDH19	*ALG8*
PI4K2A	*ALG9*
PLCB1	*AMT*
PNKP	*APTX*
PNPO	*ARFGEF2*
PTPN4	*ARG1*
SCN1A	*ARHGEF15*
SCN1B	*ARHGEF9*
SCN2A	*ARL13B*
SCN8A	*ARSA*
SLC25A22	*ARSB*
SLC2A1	*ARX*
SLC6A1	*ASAH1*
SPTAN1	*ASPA*
STXBP1	*ASPM*
SYN1	*ATIC*

SYNGAP1	*ATN1*
TRAPPC9	*ATP13A2*
UBE3A	*ATP1A2*
PTS	*ATP1A3*
GCH1	*ATP2A2*
QDPR	*ATP5A1*
PCBD1	*ATP6AP2*
SPR	*ATP6V0A2*
TH	*ATP6V1B1*
DDC	*ATP7A*
SLC6A3	*ATPAF2*
SLC18A2	*ATR*
SLC18A1	*ATRX*
SLC2A1	*B4GALT1*
DHFR	*BCKDHA*
FOLR1	*BCKDHB*
PNPO	*BCKDK*
ALDH7A1	*BCS1L*
ALDH5A1	*BRAF*
SLC6A8	*BRAT1*
GAMT	*BRD2*
GATM	*BSND*
	BTD
	BUB1B
	C12orf57
	C12orf65
	CA2
	CACNA1A
	CACNAIH
	CACNA2D1
	CACNB4
	CASK
	CASR
	CBL
	CC2D2A
	CCDC88C
	CCL2
	CDK5RAP2
	CDKL5
	CDON
	CELSR1
	CENPJ
	CEP152
	CEP290
	CERS1

	CHD2
	CHRNA2
	CHRNA4
	CHRNA7
	CHRNB2
	CLCN2
	CLCN4
	CLCNKA
	CLCNKB
	CLDN16
	CLDN19
	CLN3
	CLN5
	CLN6
	CLN8
	CNR1
	CNR2
	CNTN2
	CNTNAP2
	COG1
	COG4
	COG5
	COG6
	OG7
	OG8
	COL18A1
	COL4A1
	COQ2
	COQ9
	COX1O
	COX15
	CPA6
	CPT1A
	CPT2
	CREBBP
	CRH
	CSNK1G1
	CSTB
	CTSA
	CTSD
	CTSF
	CUL4B
	CYP27A1
	CYP2C19
	CYP2C9

	CYP3A4
	CYP3A5
	DAGLA
	DBT
	DCLK2
	DCX
	DDC
	DDOST
	DEPDC5
	DHCR7
	DLD
	DLGAP2
	DNAJC5
	DNM1
	DOCK7
	DOLK
	DPAGT1
	DPM1
	DPM3
	DPYD
	DRD2
	DYNC1H1
	DYRKIA
	EEF1A2
	EFHC1
	FHC2
	EHMTI
	EIF2B1
	EIF2B2
	EIF2B3
	EIF2B4
	EIF2B5
	ELP4
	EMX2
	EOMES
	EP300
	EPM2A
	ETFA
	ETFB
	ETFDH
	EXOSC3
	FAAH
	FASN
	FGD1
	FGF8

	FGFR3
	FH
	FKRP
	FKTN
	FLNA
	FLVCR2
	FOLR1
	FOXG1
	FOXH1
	FUCA1
	FXYD2
	GABBR1
	GABBR2
	GABRA1
	GABRA2
	GABRA3
	GABRA4
	GABRA5
	GABRA6
	GABRB1
	GABRB2
	GABRB3
	GABRD
	GABRE
	GABRG1
	GABRG2
	GABRG3
	GABRP
	GABRQ
	GABRR1
	GABBR2
	GABRR3
	GALC
	GALNS
	GAMT
	GATAD2B
	GATM
	GCDH
	GOSH
	GFAP
	GJD2
	GLB17
	GLDC
	GLI2
	GLI3

	GLRA1
	GLRB
	GLUD1
	GLUL
	GNAO1
	GNE
	GNPTAB
	GNPTG
	GNS
	GOSR2
	GPC3
	GPHN
	GPR55
	GRIA3
	GRIN1
	GRIN2A
	GRIN2B
	GRN
	GUSB
	HCN1
	HCN2
	HCN3
	HCN4
	HERC2
	HEXA
	HEXB
	HGSNAT
	HNRNPU
	HPD
	HRAS
	HSD11B2
	HSD17B10
	HSD17B4
	HYAL1
	IDH2
	IDS
	IDUA
	IER3IP1
	INPP5E
	IQSEC2
	JRK
	KANSL1
	KAT6B
	KCNA1
	KCNA2

	KCNAB1
	KCNB1
	KCNC1
	KCNH5
	KCNJ1
	KCNJ10
	KCNJ11
	KCNMA1
	KCNQ2
	KCNQ3
	KCNT1
	KCNV2
	KCTD7
	KDM5C
	KIAAl279
	KIAA2022
	KLHL3
	KMT2D
	KRAS
	L2HGDH
	LAMA2
	LARGE
	LBR
	LG11
	LIAS
	LIG4
	LRPPRC
	MAGI2
	MAGT1
	MAP2K1
	MAP2K2
	MAPK10
	MBD5
	MCOLN1
	MCPH1
	ME2
	MECP2
	MED12
	MED17
	MEF2C
	MFSD8
	MGAT2
	MGLL
	MGME1
	MLC1

	MMACHC
	MOCS1
	MOCS2
	MOOS
	MPDU1
	MPI
	THFR
	MTOR
	MTR
	MTRR
	NAGLU
	NALCN
	NDE1
	NDUFAll
	NDUFA2
	NDUFAF6
	NDUFS1
	NDUFS3
	NDUFS4
	NDUFS7
	NDUFS8
	NDUFV1
	NECAP1
	NEDD4L
	NEU1
	NF1
	NGLY1
	NHEJ1
	NHLRCI
	NIPBL
	NODAL
	NOL3
	NOTCH3
	NPC1
	NPC2
	NPHP1
	NR2F1
	NR3C2
	NRAS
	NRXN1
	OFD1
	OPA1
	OPHN1
	PAFAH1B1
	PAK3

	PANK2
	PAX6
	PC
	PCDH19
	PCNT
	PDHA1
	PDHX
	PDSS1
	PDSS2
	PEX1
	PEX10
	PEX12
	PEX13
	PEX14
	PEX16
	PEX19
	PEX2
	PEX26
	PEX3
	PEX5
	PEX6
	PEX7
	P0K1
	PGM1
	PHF6
	PHGDH
	PIGA
	PIGO
	PIGQ
	P1GV
	PIK3CA
	P1K3R2
	PLA2G6
	PLCB1
	PLP1
	PMM2
	PNKP
	PNPO
	POLL
	POMGNT1
	POMT1
	POMT2
	PPT1
	PQBP1
	PRICKLE1

	PRICKLE2
	PRODH
	PRRT2
	PSAP
	PSAT1
	PTCH1
	PTPN11
	PURA
	OARS
	QDPR
	RAB39B
	RAB3GAP1
	RAF1
	RAI1
	RARS2
	RBFOX1
	RELN
	RFT1
	RNASEH2A
	RNASEH2B
	RNASEH2C
	RPGRIP1L
	RTTN7
	RYR3
	SAMHD1
	SCARB2
	SCN10A
	SCN11A
	SCN1A
	SCN1B
	SCN2A
	SCN2B
	SCN3A
	SCN3B
	SCN4A
	SCN4B
	SCN5A
	SCN7A
	SCN8A
	SCN9A
	SCNN1A
	SCNN1B
	SCNN1G
	SCO2
	SDHA

	SERPINI1
	SETBP1
	SGCE
	SGSH
	SHH
	SHOC2
	ST3GAL5
	SIK1
	SIX3
	SLC12A1
	SLC12A2
	SLC12A3
	SLC13A57
	SLC16A2
	SLC17A5
	SLC19A3
	SLC1A3
	SLC25A15
	SLC25A19
	SLC25A22
	SLC2A1
	SLC35A1
	SLC35A2
	SLC35C1
	SLC46A1
	SLC4A1
	SLC4A10
	SLC4A4
	SLC6A1
	SLC6A4
	SLC6A5
	SLC6A8
	SLC9A6
	SMARCA2
	SMC1A
	SMC3
	SMPD1
	SMS
	SNAP25
	SNAP29
	SNIP1
	SOS1
	SPREDI
	SPTAN1
	SRD5A3

	SRPX2
	ST3GAL3
	STIL
	STRADA
	STX1B
	STXBP1
	SUCLA2
	SUMF1
	SUOX
	SURF1
	SYN1
	SYNGAP1
	SYNJ1
	SYP
	SZT2
	TACO1
	TBC1D24
	TBCE
	TBL1XR1
	TBX1
	TCF4
	TGIF1
	TMEM165
	TMEM216
	TMEM67
	TMEM70
	TPP1
	TREX1
	TRPM6
	TSC1
	TSC2
	TSEN2
	TSEN34
	TSEN54
	TUBA1A
	TUBA8
	TUBB2A
	TUBB2B
	TUSC3
	UBE2A
	UBE3A
	VANGL1
	VDAC1
	VPS13A
	VPS13B

	VRK1
	WDR45
	WDR62
	WNK1
	WNK4
	WWOX
	ZEB2
	Z1C2

Table S2. Pathogenic mutations detected by SS, HCP, TSO and WES. Pathogenic mutations are separated by the method used: SS, Sanger Sequencing; HCP, Haloplex Custom Panel; TSO, TrueSigthOne; WES, Whole Exome Sequencing.

Num. Patients	Gene	OMIM number	Transcript	Genotype	Type of seq. Change	cDNAchange	Proteinchange	Rs number
**Potentially pathogenic mutation detected by SS**								
2	CDKL5	300203	NM_003159	Heterozygous	Nonsense	c.1675C>T	p.Arg559Ter	rs267608395
1	CDKL5	300203	NM_003159	Heterozygous	Missense	c.533G>A	p.Arg178Gln	rs267606715
1	CDKL5	300203	NM_003159	Heterozygous	Frameshift insertion	c.937insA	p.Arg313LysfsTer13	-
1	CDKL5	300203	NM_003159	Heterozygous	Missense	c.1266C>A	p.Asp422Glu	-
1	CDKL5	300203	NM_003159	Heterozygous	Frameshift deletion	c.594delC	p.Cys199ValfsTer29	-
1	CDKL5	300203	NM_003159	Heterozygous	Missense	c.872G>A	p.Cys291Tyr	rs267606714
1	CDKL5	300203	NM_003159	Heterozygous	Nonsense	c.2704C>T	p.Gln902Ter	rs786204981
1	CDKL5	300203	NM_003159	Heterozygous	Frameshift insertion	c.1415insA	p.Ile473AsnfsTer6	rs786204970
1	CDKL5	300203	NM_003159	Heterozygous	Frameshift deletion	c.1341delC	p.Phe447LeufsTer46	rs786204968
1	CDKL5	300203	NM_003159	Heterozygous	Frameshift deletion	c.1549delT	p.Phe517SerfsTer6	rs786204972
1	CDKL5	300203	NM_003159	Heterozygous	Frameshift deletion	c.1533delC	p.Ser512ValfsTer11	-
1	CDKL5	300203	NM_003159	Heterozygous	Missense	c.528G>T	p.Trp176Cys	rs786204989
1	CDKL5	300203	NM_003159	Heterozygous	Nonsense	c.858C>A	p.Tyr286Ter	-
1	CDKL5	300203	NM_003159	Heterozygous	Frameshift deletion	c.2111delA	p.Tyr704SerfsTer80	-
1	FOXG1	164874	NM_005249	Heterozygous	Missense	c.688C>G	p.Arg230Gly	-
1	FOXG1	164874	NM_005249	Heterozygous	Frameshift insertion	c.461insG	p.Glu155GlyfsTer300	-
1	FOXG1	164874	NM_005249	Heterozygous	Frameshift deletion	c.292_295delGGCC	p.Gly98ProfsTer93	-
1	FOXG1	164874	NM_005249	Heterozygous	Missense	c.693C>A	p.Hys231Gln	-
1	FOXG1	164874	NM_005249	Heterozygous	Missense	c.619A>T	p.Ile207Phe	-

1	FOXG1	164874	NM_005249	Heterozygous	Missense	c.541A>G	p.Lys181Glu	-
1	FOXG1	164874	NM_005249	Heterozygous	Frameshift deletion	c.201_224del24	p.Pro66_Gln73del	-
1	FOXG1	164874	NM_005249	Heterozygous	Stop loss	c.1470A>C	p.Ter490TyrextTer21	-
1	FOXG1	164874	NM_005249	Heterozygous	Nonsense	c.624C>G	p.Tyr208Ter	rs267606826
42	MECP2	300005	NM_004992	Heterozygous	Nonsense	c.763C>T	p.Arg255Ter	rs61749721
37	MECP2	300005	NM_004992	Heterozygous	Nonsense	c.502C>T	p.Arg168Ter	rs61748421
35	MECP2	300005	NM_004992	Heterozygous	Missense	c.473C>T	p.Thr158Met	rs28934906
27	MECP2	300005	NM_004992	Heterozygous	Nonsense	c.808C>T	p.Arg270Ter	rs61751362
27	MECP2	300005	NM_004992	Heterozygous	Missense	c.916C>T	p.Arg306Cys	rs28935468
21	MECP2	300005	NM_004992	Heterozygous	Nonsense	c.880C>T	p.Arg294Ter	rs61751362
14	MECP2	300005	NM_004992	Heterozygous	Missense	c.397C>T	p.Arg133Cys	rs28934904
13	MECP2	300005	NM_004992	Heterozygous	Frameshift deletion	c.806delG	p.Gly269AlafsTer20	rs61750241
8	MECP2	300005	NM_004992	Heterozygous	Missense	c.316C>T	p.Arg106Trp	rs28934907
8	MECP2	300005	NM_004992	Heterozygous	Missense	c.455C>G	p.Pro152Arg	rs61748404
7	MECP2	300005	NM_004992	Heterozygous	Frameshift deletion	c.1164_1207del44	p.Pro389Ter	rs63749749
4	MECP2	300005	NM_004992	Heterozygous	Frameshift deletion	c.1157_1200del44	p.Leu386GlnfsTer4	rs63749748
3	MECP2	300005	NM_004992	Heterozygous	Missense	c.965C>T	p.Pro322Leu	rs61751450
3	MECP2	300005	NM_004992	Heterozygous	Nonsense	c.423C>G	p.Tyr141Ter	rs61748396
2	MECP2	300005	NM_004992	Heterozygous	Missense	c.468C>G	p.Asp156Glu	rs61748408
2	MECP2	300005	NM_004992	Heterozygous	Frameshift insertion	c.710insG	p.Gly238TrpfsTer21	rs267608517
2	MECP2	300005	NM_004992	Heterozygous	Frameshift insertion	c.710delG	p.Gly238ValfsTer11	rs61749743
2	MECP2	300005	NM_004992	Heterozygous	Missense	c.380C>T	p.Pro127Leu	rs267608387
2	MECP2	300005	NM_004992	Heterozygous	Missense	c.905C>G	p.Pro302Arg	rs61749723
2	MECP2	300005	NM_004992	Heterozygous	Frameshift deletion	c.1163_1197del35	p.Pro388HisfsTer5	rs267608599
1	MECP2	300005	NM_004992	Heterozygous	Frameshift deletion	c.787delC	p.Ala263Pro_fsTer26	-
1	MECP2	300005	NM_004992	Heterozygous	Frameshift indel	c.831_966delins136	p.Ala278PhefsTer15	-
1	MECP2	300005	NM_004992	Heterozygous	Missense	c.317G>A	p.Arg106Gln	rs61754457

1	MECP2	300005	NM_004992	Heterozygous	Missense	c.317G>T	p.Arg106Leu	rs61754457
1	MECP2	300005	NM_004992	Heterozygous	Missense	c.332G>A	p.Arg111Lys	-
1	MECP2	300005	NM_004992	Heterozygous	Missense	c.398G>A	p.Arg133His	rs61748389
1	MECP2	300005	NM_004992	Heterozygous	Frameshift deletion	c.591delG	p.Arg198AspfsTer12	-
1	MECP2	300005	NM_004992	Heterozygous	Frameshift indel	c.748_750delinsCG	p.Arg250AlafsTer39	-
1	MECP2	300005	NM_004992	Heterozygous	Frameshift insertion	c.755_1063del309	p.Arg253_Ser355	-
1	MECP2	300005	NM_004992	Heterozygous	Frameshift insertion	c.453ins7	p.Arg253PhefsTer8	-
1	MECP2	300005	NM_004992	Heterozygous	Frameshift deletion	c.808delC	p.Arg270GlufsTer19	rs62931162
1	MECP2	300005	NM_004992	Heterozygous	Missense	c.917G>A	p.Arg306His	rs61751443
1	MECP2	300005	NM_004992	Heterozygous	Missense	c.925C>T	p.Arg309Trp	rs61751444
1	MECP2	300005	NM_004992	Heterozygous	Frameshift deletion	c.1061_1166del96	p.Arg354LeufsTer20	-
1	MECP2	300005	NM_004992	Heterozygous	Frameshift deletion	c.100_103delGATA	p.Asp34ArgfsTer89	rs61754428
1	MECP2	300005	NM_004992	Heterozygous	Frameshift deletion	c.1280_1335del56	p.Asp427GlyfsTer41	-
1	MECP2	300005	NM_004992	Heterozygous	Frameshift insertion	c.621dupG	p.Gln208AlafsTer28	-
1	MECP2	300005	NM_004992	Heterozygous	Nonsense	c.622C>T	p.Gln208Ter	rs61749729
1	MECP2	300005	NM_004992	Heterozygous	Nonsense	c.730C>T	p.Gln244Ter	rs61749747
1	MECP2	300005	NM_004992	Heterozygous	Nonsense	c.139C>T	p.Gln47Ter	-
1	MECP2	300005	NM_004992	Heterozygous	Missense	c.410A>G	p.Glu137Gly	rs61748392
1	MECP2	300005	NM_004992	Heterozygous	Frameshift deletion	c.869_915del47	p.Glu290AlafsTer25	-
1	MECP2	300005	NM_004992	Heterozygous	Frameshift deletion	c.1041_4447del3407	p.Glu348LeufsTer21	-
1	MECP2	300005	NM_004992	Heterozygous	Nonsense	c.1189G>T	p.Glu397Ter	rs56268439
1	MECP2	300005	NM_004992	Heterozygous	Frameshift deletion	c.189_190delGA	p.Glu63AspfsTer27	rs61754436
1	MECP2	300005	NM_004992	Heterozygous	Missense	c.308G>T	p.Gly103Val	-
1	MECP2	300005	NM_004992	Heterozygous	Frameshift deletion	c.651_1137del486	p.Gly218CysfsTer29	-
1	MECP2	300005	NM_004992	Heterozygous	Frameshift insertion	c.704insG	p.Gly238TrpfsTer21	-

1	MECP2	300005	NM_004992	Heterozygous	Indel rearrangement	c.(754_773insdelAGCGTTT)(873_997insdel51)(1025_1063insdelCGG)1075 T>C(1090_1128del39)(1149_1194del46)	p.Gly252SerfsTer32	-
1	MECP2	300005	NM_004992	Heterozygous	Frameshift deletion	c.1115_1190del76	p.His372ArgfsTer12	-
1	MECP2	300005	NM_004992	Heterozygous	Missense	c.414G>C	p.Leu138Phe	-
1	MECP2	300005	NM_004992	Heterozygous	Frameshift deletion	c.445_901del456	p.Leu150ProfsTer19	-
1	MECP2	300005	NM_004992	Heterozygous	Frameshift deletion	c.1150_1195del46	p.Leu386AlafsTer8	-
1	MECP2	300005	NM_004992	Heterozygous	Frameshift deletion	c.1157_1188del32	p.Leu386ArgfsTer8	rs267608585
1	MECP2	300005	NM_004992	Heterozygous	Frameshift deletion	c.1157_1197del41	p.Leu386HisfsTer5	rs267608327
1	MECP2	300005	NM_004992	Heterozygous	Frameshift deletion	c.1157_3664del2508	p.Leu386LeufsTer20	-
1	MECP2	300005	NM_004992	Heterozygous	Nonsense	c.598A>T	p.Lys200Ter	rs61749718
1	MECP2	300005	NM_004992	Heterozygous	Frameshift insertion	c.691insG	p.Lys233GlnfsTer3	-
1	MECP2	300005	NM_004992	Heterozygous	Frameshift insertion	c.69_70insT	p.Lys24HisfsTer8	-
1	MECP2	300005	NM_004992	Heterozygous	Nonsense	c.766A>T	p.Lys256Ter	rs786205027
1	MECP2	300005	NM_004992	Heterozygous	Frameshift deletion	c.856_859delAAAG	p.Lys286ProfsTer2	rs61750256
1	MECP2	300005	NM_004992	Heterozygous	Missense	c.914A>G	p.Lys305Arg	rs61751441
1	MECP2	300005	NM_004992	Heterozygous	Frameshift indel	c.961_1188delins220	p.Lys321GlyfsTer6	-
1	MECP2	300005	NM_004992	Heterozygous	Frameshift deletion	c.1009_1016del8	p.Lys337Ter	-
1	MECP2	300005	NM_004992	Heterozygous	Frameshift deletion	c.1054_1083del30	p.Lys352_Pro362del	-
1	MECP2	300005	NM_004992	Heterozygous	Frameshift deletion	c.116_119delAAGA	p.Lys39ArgfsTer85	-
1	MECP2	300005	NM_001110792.1	Heterozygous	Start loss	c.1A>G	p.Met1Val	-
1	MECP2	300005	NM_004992	Heterozygous	Missense	c.674C>T	p.Pro225Arg	rs61749715
1	MECP2	300005	NM_004992	Heterozygous	Frameshift deletion	c.687_696del10	p.Pro230ArgfsTer15	-
1	MECP2	300005	NM_004992	Heterozygous	Missense	c.905C>A	p.Pro302His	rs61749723
1	MECP2	300005	NM_004992	Heterozygous	Missense	c.904C>T	p.Pro302Ser	rs61751373
1	MECP2	300005	NM_004992	Heterozygous	Frameshift deletion	c.1150_1177del28	p.Pro385ArgfsTer15	-
1	MECP2	300005	NM_004992	Heterozygous	Frameshift deletion	c.1152_1195del44	p.Pro385HisfsTer5	rs267608372

1	MECP2	300005	NM_004992	Heterozygous	Frameshift deletion	c.1153_2387del1235	p.Pro385ValfsTer51	-
1	MECP2	300005	NM_004992	Heterozygous	Frameshift deletion	c.1159_1210del52	p.Pro387SerfsTer5	-
1	MECP2	300005	NM_004992	Heterozygous	Frameshift insertion	c.1164_4665del3502insC	p.Pro388ProfsTer1	-
1	MECP2	300005	NM_004992	Heterozygous	Frameshift deletion	c.1162_4991del3580	p.Pro389GlnfsTer90	-
1	MECP2	300005	NM_004992	Heterozygous	In-frame deletion	c.276_281del4	p.Pro93_Tyr95del	-
1	MECP2	300005	NM_004992	Heterozygous	Missense	c.401C>G	p.Ser134Cys	rs61748390
1	MECP2	300005	NM_004992	Heterozygous	Frameshift insertion	c.869insG	p.Ser291ValfsTer40	-
1	MECP2	300005	NM_004992	Heterozygous	Nonsense	c.203C>G	p.Ser68Ter	rs267608438
1	MECP2	300005	NM_004992	Heterozygous	Frameshift deletion	c.236delC	p.Ser80ProfsTer45	-
1	MECP2	300005	NM_004992	Heterozygous	Stop loss	c.1459T>G	p.Ter487Gly_extTer27	-
1	MECP2	300005	NM_004992	Heterozygous	Frameshift deletion	c.433_769del337	p.Val145LeufsTer32	-
1	MECP2	300005	NM_004992	Heterozygous	Frameshift deletion	c.46delC	p.Val31Ter	-
1	MECP2	300005	NM_004992	Heterozygous	Frameshift deletion	c.1163_1188del26	Pro388ArgfsTer8	rs267608600
**Potentially pathogenic mutation detected by HCP**								
1	CDKL5	300203	NM_003159	Heterozygous	Missense	c.1906G>T	p.Ala636Ser	-
1	CDKL5	300203	NM_003159	Heterozygous	Splicing variant	c.65-2A>G	Miss-splicing	-
1	CDKL5	300203	NM_003159	Heterozygous	Missense	c.65G>A	p.Gly22Glu	-
1	CDKL5	300203	NM_003159	Heterozygous	Splicing variant	c.825+1G>A	Miss-splicing	-
1	FOXG1	164874	NM_005249	Heterozygous	Frameshift insertion	c.1117_1118insCGTA	p.Ala375ValfsTer81	-
1	FOXG1	164874	NM_005249	Heterozygous	Missense	c.653A>C	p.Tyr218Ser	-
1	FOXG1	164874	NM_005249	Heterozygous	Frameshift insertion	c.953_954insC	p.Arg320ProfsTer135	-
1	KCNQ2	602235	NM_172107	Heterozygous	Missense	c.593G>A	p.Arg198Gln	rs796052621
1	KCNQ2	602235	NM_172107	Heterozygous	Missense	c.637C>T	p.Arg213Trp	rs118192203
3	MECP2	300005	NM_004992	Heterozygous	Missense	c.473C>T	p.Thr158Met	rs28934906
3	MECP2	300005	NM_004992	Heterozygous	Missense	c.916C>T	p.Arg306Cys	rs28935468
2	MECP2	300005	NM_004992	Heterozygous	Missense	c.455C>G	p.Pro152Arg	rs61748404
2	MECP2	300005	NM_004992	Heterozygous	Nonsense	c.808C>T	p.Arg270Ter	rs61751362

1	MECP2	300005	NM_004992	Hemizygous	Frameshift insertion	c.397insT	p.Arg133LeufsTer3	-
1	MECP2	300005	NM_004992	Heterozygous	Missense	c.403A>G	p.Lys135Glu	rs61748391
1	MECP2	300005	NM_004992	Heterozygous	Missense	c.468C>G	p.Asp156Glu	rs61748408
1	MECP2	300005	NM_004992	Heterozygous	Nonsense	c.502C>T	p.Arg168Ter	rs61748421
1	MECP2	300005	NM_004992	Hemizygous	In-frame deletion	c.651_1137del486	p.Pro207_His369	-
1	MECP2	300005	NM_004992	Heterozygous	Nonsense	c.763C>T	p.Arg255Ter	rs61749721
1	MECP2	300005	NM_004992	Heterozygous	Nonsense	c.880C>T	p.Arg294Ter	rs61751362
1	MECP2	300005	NM_004992	Heterozygous	Missense	c.905C>T	p.Pro302Leu	rs61749723
2	STXBP1	602926	NM_003165	Heterozygous	Missense	c.874C>T	p.Arg292Cys	-
2	STXBP1	602926	NM_003165	Heterozygous	Missense	c.875G>A	p.Arg292His	rs796053361
1	STXBP1	602926	NM_003165	Heterozygous	Missense	c.1216C>T	p.Arg406Cys	rs796053367
1	STXBP1	602926	NM_003165	Heterozygous	In-frame deletion	c.124_126delTCC	p.Ser42del	-
1	STXBP1	602926	NM_003165	Heterozygous	Splicing variant	c.326-3C>G	Miss-splicing	-
1	STXBP1	602926	NM_003165	Heterozygous	Missense	c.704G>A	p.Arg235Gln	-
1	TCF4	602272	NM_001243236	Heterozygous	In-frame indel	c.1169_1175delTAGAAAGinsAAA	p.Leu390Ter	-
1	TCF4	602272	NM_001243236	Heterozygous	Missense	c.1733G>A	p.Arg578His	rs121909123
1	TCF4	602272	NM_001243236	Heterozygous	Nonsense	c.1774C>T	p.Gln592Ter	-
1	TCF4	602272	NM_001243236	Heterozygous	Frameshift deletion	c.514_517delAAAG	p.Lys172PhefsTer61	rs398123561
**Potentially pathogenic mutation detected by TSO**								
1	-	-	-	Heterozygous	Gross deletion	Xp11.22(52954520-53394275)x1	-	-
1	-	-	-	Heterozygous	Gross deletion	15q11.2-q13.1 (22756650-29209680)x1	-	-
1	-	-	-	Heterozygous	Gross duplication	14q32.11-q32.33(90949120-107287505)x3	-	-
1	CDKL5	300203	NM_003159	Heterozygous	Frameshift deletion	c.560_571delCCTATGGAAAGT	p.Tyr188_Ser191del	-
1	CDKL5	300203	NM_003159	Heterozygous	Splicing variant	c.99+1G>T	Miss-splicing	rs267608421
1	MECP2	300005	NM_004992	Heterozygous	Frameshift deletion	c.528delC	p.Lys177AsnfsTer33	-
1	MEF2C	600662	NM_001193347	Heterozygous	Missense	c.48C>G	p.Asn16Lys	-
1	MEF2C	600662	NM_001193347	Heterozygous	Frameshift deletion	c.989_990delGT	p.Gly330AspfsTer7	-

1	SCN2A	182390	NM_001040142	Heterozygous	Missense	c.3631G>A	p.Glu1211Lys	rs387906684
1	SCN2A	182390	NM_001040142	Heterozygous	Missense	c.5317G>A	p.Ala1773Thr	-
1	SYNGAP1	603384	NM_006772	Heterozygous	Frameshift deletion	c.2019delA	p.Thr674ProfsTer36	-
1	SYNGAP1	603384	NM_006772	Heterozygous	Frameshift deletion	c.1782delC	p.Leu595CysfsTer55	rs587780470
**Potentially pathogenic mutation detected by WES**								
1	CACNA1I	608230	NM_021096	Heterozygous	Missense	c.4435C>T	p.Leu1479Phe	-
1	CDKL5	300203	NM_003159	Heterozygous	Missense	c.587C>G	p.Ser196Trp	-
1	CHRNA5	118505	NM_000745	Heterozygous	Missense	c.748C>A	p.Pro250Thr	-
1	GABBR2	607340	NM_005458	Heterozygous	Missense	c.1699G>A	p.Ala567Thr	-
1	GRIN2B	138252	NM_000834	Heterozygous	Missense	c.1657C>A	p.Pro553Thr	-
1	HCN1	602780	NM_021072	Heterozygous	Missense	c.1159G>T	p.Ala387Ser	-
1	MECP2	300005	NM_004992	Heterozygous	Missense	c.674C>G	p.Pro225Arg	rs61749715
1	SCN1A	182389	NM_001165963	Heterozygous	Missense	c.3965G>C	p.Arg1322Thr	-
1	SLC6A1	137165	NM_003042	Heterozygous	Missense	c.919G>A	p.Gly307Arg	-
1	TCF4	602272	NM_001083962	Heterozygous	Frameshift deletion	c.1438delC	p.Gln480SerfsTer8	-

Table S3. Rett-like gene list. 17 Rett-like gene list used for statistical and bioinformatics data file: clinical association, phenotype MIM number and gene are included.

Clinical association	Phenotype MIM number	Gene
Rett syndrome	312750	*MECP2*
Rett syndrome, early epilepsy variant	312750	*CDKL5*
Rett syndrome, congenital variant	613454	*FOXG1*
Pitt-Hopkins syndrome	610954	*TCF4*
Mental retardation, stereotypic movements, epilepsy, and/or cerebral malformations	613443	*MEF2C*
Rett-like/Angelman-like	105830	*UBE3A*
Mental retardation, autosomal recessive 38	615516	*HERC2*
Epileptic encephalopathy, early infantile, 1	308350	*ARX*
Candidate disease gene for RTT	N.A.	*NTNG1*
Epileptic encephalopathy, early infantile, 7	613720	*KCNQ2*
Pelizaeus-Merzbacher disease	312080	*PLP1*
Epileptic encephalopathy, early infantile, 11	613721	*SCN2A*
Phelan-McDermid syndrome	606232	*SHANK3*
Gamma-hydroxibutyric aciduria	271980	*ALDH5A1*
Epileptic encephalopathy, early infantile, 4	612164	*STXBP1*
Clinic modulator in Rett syndrome	N.A.	*BDNF*
Speech-language disorder-1	602081	*FOXP2*

Table S4. Primers list used for validation. Gene list, primer sequences and genomic position (GRCh37/hg19) are included.

Gene	Primers	Sequence	Genomic position (GRCh37/hg19)
MECP2	F	CACTGTGTGTTACGTGCCAGT	chrX:153357476-153357940
	R	GGCACAGTTTGGCACAGTTAT	
MECP2	F	CCCTGGGCACATACATTTTC	chrX:153297571+153298157
	R	TCTCTGTTGTCCTGGGGAAG	
MECP2	F	GGCAGTGTGACTCTCGTTCA	chrX:153296272-153297030
	R	AGTCCTTTCCCGCTCTTCTC	
MECP2	F	GAGACCGTACTCCCCATCAA	chrX:153295649-153296387
	R	CCAACTACTCCCACCCTGAA	
CDKL5	F	CCCTGCTGCTCTGATTTTTC	chrX:18460134+18460644
	R	GAAACTCCGCCTCAAAACAA	
CDKL5	F	CCTCATTGGCTCCTACCAGA	chrX:18443569+18443907
	R	GTGGGTCTCCACTCTCAGGA	
CDKL5	F	taagattggttactagAgtactgc	chrX:18524988+18525399
	R	GACACACATGTGAATTGATATAGGTT	
CDKL5	F	gagaagcaatgtcagtatagcag	chrX:18528865+18529058
	R	CATGCCCACACGCAAAGACCA	
CDKL5	F	caactggaatccccagtcgga	chrX:18582531+18582751
	R	AGTGTCTGACCAGCTAGATCCCACTTCT	
CDKL5	F	gaagtactcaaagcagaaggtga	chrX:18593362+18593646
	R	TCGGGCAAATGTGCACATTGGCA	
CDKL5	F	gctctgtattggatgaattattctag	chrX:18597866+18598162
	R	GACAGTAACATGTGAAATACTCTTAACTT	
CDKL5	F	cagtgtcaatcaggagaacatag	chrX:18599949+18600111
	R	TAATTCTGTAAGTACCAGGACTTA	
CDKL5	F	gcccatgcgagaacagtcattac	chrX:18602255+18602534
	R	GCAAATGACAATAGAATCAGCAG	
CDKL5	F	agttgccaaaataatctcttcctt	chrX:18606040+18606323
	R	GAACAATGACTCAAATACTGCAG	
CDKL5	F	aacactcacaagcacgtgca	chrX:18613397+18613669
	R	TTATTTGCCATTCCACATCTCCT	
CDKL5	F	gactttgtaatgttcttaacgatc	chrX:18616539+18616819
	R	CTAATTGCATCATTTAAGCAGCC	
CDKL5	F	ttgtgtgtcagctattgaggg	chrX:18621969+18622252
	R	GGTATGTTGTTGTTGGTGAGATC	
CDKL5	F	TGCACACCAAAACCTACCAAGC	chrX:18622180+18622508
	R	TCCTACAGGACCAAGGCCAAAAGC	
CDKL5	F	GAGTCGGCATAGCTATATTGACAC	chrX:18622436+18622789
	R	GAATGGCTACTGTCCATGTGC	
CDKL5	F	AACGCTGGACTCACGTCGAAC	chrX:18622703+18623020
	R	CCACCAGATTCAGTCAAGGTG	
CDKL5	F	ctggttatggtcctagttctacc	chrX:18626835+18627119
	R	GTGGGAGACTGGGTATTAATAC	

CDKL5	F	cataggcaatattgtcatcaatgtg	chrX:18627525+18627793
	R	GTGTAGGTGAGAAGGCCGCTG	
CDKL5	F	cataggcaatattgtcatcaatgtg	chrX:18627525+18627793
	R	GTGTAGGTGAGAAGGCCGCTG	
CDKL5	F	gaaaagtccatcagtgacttac	chrX:18631235+18631483
	R	GGACACTAAAAAGCTCATCCAGA	
CDKL5	F	ggctataggaacctagtgtcatg	chrX:18637880+18638161
	R	AAGTGCAAAGTGTAAAGTATCCAT	
CDKL5	F	ctcctcttgggtgtggttgc	chrX:18643170+18643481
	R	GCTCAGCCTTACTGTAACATTG	
CDKL5	F	tctaacttgaatcctgtgtgc	chrX:18646450+18646749
	R	TCCTGGTCACAGAGGACACATG	
CDKL5	F	gtgggcagaagtggccaata	chrX:18664037+18664259
	R	GTCTAGGGTCGTTATGGCAGC	
CDKL5	F	accttggcttcagctggtgtc	chrX:18668445+18668769
	R	GGGCAATTCCGAGGTACAGC	
CDKL5	F	gccagagtgcacctgctagc	chrX:18671485+18671770
	R	AAGGAAAACTCAACCTCAGCG	
FOXG1	F	CCCGTTCGGCACCCACCGGT	chr14:29236397+29237174
	R	CGGATGGAGTTCTGCCAGC	
FOXG1	F	AACGGCAAGTACGAGAAGC	chr14:29237011+29237263
	R	ACGGGTCCAGCATCCAGTA	
FOXG1	F	CCACAATCTGTCCCTCAACAA	chr14:29237175+29237525
	R	TGAGTCAACACGGAGCTGT	
FOXG1	F	TTCCTGTCCCTGCACCAC	chr14:29237401+29237774
	R	CTCTGCGAAGTCATTGAC	
FOXG1	F	CCCTGCTCTGGGACCTACTC	chr14:29237668+29237996
	R	AGTGAACGTTTTACACACAT	
STXBP1	F	tccagtctgggtgacagaat	chr9:130423240+130423524
	R	caggatgcagatttcctttg	
STXBP1	F	aactactgttcctccacactgt	chr9:130438022+130438395
	R	ctgcaacgatctgactacactg	
STXBP1	F	ggttcgatgaatgttgtggaga	chr9:130430302+130430609
	R	attacgggttagcatgttggtg	
TCF4	F	ACCTGACACCAGAGCAGAAG	chr18:52895901-52896290
	R	tacaggagaaagggatgctg	
SCN2A	F	AGGATGAAAAGATGGCACAG	chr2:166152323+166152636
	R	aaataggcagtgaaggcaact	
KCNQ2	F	CCCGCTTTGTAGACATCATGG	chr20:62075889-62076199
	R	CACTGACTCCATCCCTCCAC	
SLC6A3	F	gccccagggtttctgacta	chr5:1394672-1394910
	R	CGGCACGGAAAGGTGTAA	
STXBP1	F	gagcgagaatcacagcagat	chr9:130415938+130416188
	R	cacaggttgctggaagaatc	
STXBP1	F	cttgtgaagcatctccctgt	chr9:130428415+130428633
	R	tatttttggctcatgtgcag	

TCF4	F	aatttaggcacccctttga	chr18:52921591-52921987
	R	ctgaaatgggctcatcgtat	
TCF4	F	gaatcgaatgtgatcgtggt	chr18:53017477-53017784
	R	AGATAAACGTGCTGCTCTGG	
SLC2A1	F	cactctgagccaccctca	chr1:43395198-43395490
	R	gttcaccatgcacacttgac	
MEF2C	F	CAGTCATTGGCTACCCCAGT	chr5:88024903-88025134
	R	ggtaatgctgcagtgctgtg	
MEF2C	F	tgacttgctgttagagtggtatt	chr5:88119409-88119791
	R	cagcactttaactggtcacatt	
SCN2A	F	ACAGCATGATCTGCCTGTTC	chr2:166245421+166245838
	R	GCAATGAGCTGGACTTTGTT	
SCN2A	F	tgtaaatgaatctcccaccaac	chr2:166223646+166223920
	R	tcagatgaaaggtgaaggagtg	
SYNGAP1	F	ccggtctctctctttctctctc	chr6:33440675+33441177
	R	TAAACctagccaagaatgcaga	
SYNGAP1	F	GTCCCCAGAGATCCTGAGAT	chr6:33441052+33441487
	R	GTGGTGACACCTCGAGAAAC	
HCN1	F	CAGCAGACTGTTTCCACTTCA	chr5:45396198-45397021
	R	CATGCACAATAGCTGCCTGT	
TCF4	F	TCAGCGCCCTCTAGTGAAAC	chr18:52901609-52902050
	R	TTAGCGGGCGAAGTTCTAAA	
GRIN2B	F	TACAATCTAACCTAGGCCCTGG	chr12:13764558-13764935
	R	TGGATATGCTAGGGAAAATGCAG	
CACNA1I	F	CCACTGCCAACCTGAGTGA	chr22:40066439+40067235
	R	CACAGTCATTGCCACCCATG	
CHRNA5	F	GAGCAGGGTCCCTATGTAGC	chr15:78881983+78882780
	R	CGCCATGGCATTATGTGTTGA	
GABBR2	F	GTGACCTGGGTCTGGTAAGTG	chr9:101133405-101134056
	R	TTCCTTTGACAAGGTCCCCAG	
SLC6A1	F	CTGTCTGACTCCGAGGTGAG	chr3:11067255+11067936
	R	GACGATGATGGAGTCCCTGA	
SCN1A	F	ttttgtgtgtgcagGTTTCATT	chr6:33441052+33441487
	R	aggcctatttctcttgcatatca	

Figure S1. Work-flow in molecular diagnosis 1999-2017. This figure shows how the work flow was followed and how the new techniques were introduced during these years. In brackets, number of samples analyzed in each technique, number without bracket are negative samples used using another technique; in red, Sanger Sequencing; in purple, Whole exome sequencing; in blue, Haloplex Custom panel; in green, TruSigth One panel

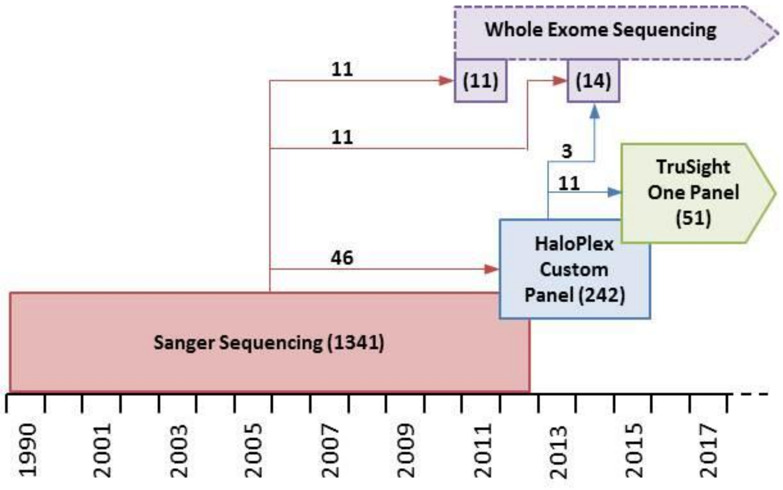

Supplement: Supplementary file 1 — Supplementary files [file 41598_2017_11620_MOESM1_ESM.pdf]
